# Stay-on-Task Exercises as a Tool To Maintain Focus during a CRISPR CURE

**DOI:** 10.1128/jmbe.00114-21

**Published:** 2021-06-30

**Authors:** Ben A. Evans, Ethan S. Pickerill, Douglas A. Bernstein

**Affiliations:** a Department of Biology, Ball State University, Muncie, Indiana, USA

**Keywords:** CRISPR, genome editing, CURE, stay-on-task exercise, CRISPR laboratory exercise

## Abstract

Course-based undergraduate research experiences (CURE) offer the chance for students to experience authentic research investigation in a classroom setting. Such hands-on experiences afford unique opportunities work on a semi-independent research project in an efficient, structured environment. We have developed a CRISPR CURE in which undergraduate and graduate students use *in silico*, *in vitro*, and *in vivo* techniques to edit a fungal genome. During the development of this course, we have found that the asynchronous nature of the CRISPR CURE activities can be disruptive and lead to unproductive class time. To overcome this challenge, we have developed stay-on-task exercises (SOTEs). These short low-stakes assessments provide structured activities that are performed during these asynchronous incubation periods. SOTE activities leverage potentially unproductive class time and complement the CURE learning objectives. We have found SOTEs to be one method of maintaining classroom structure during a CURE. Furthermore, SOTE complexity, length, and subject can be easily modified to match course learning objectives.

## INTRODUCTION

Course-based undergraduate research experiences (CUREs) provide opportunities for students to gain valuable lab or field experience performing guided independent research during a course (1). CUREs allow multiple students to concurrently investigate portions of important scientific problems. In addition, many CURE courses leverage well-established techniques so students have the best possible chance of collecting interpretable data. Exercises that introduce molecular biology techniques are quite practical, as they teach students transferrable skills that can be applied by the students when they enter the workforce or graduate school (2, 3).

As with all instructional techniques, CUREs come with challenges (4). Given the relatively short period of investigation and inexperience of the investigators, clarity and swiftness by which results can be obtained take on added importance compared to the case with more senior lead investigations. Another challenge inherent in molecular biology CUREs is that such techniques often require incubation periods long enough to be performed in a typical class period. As the semester progresses, it is unlikely that every student will be working on the same step of the project, and consequently, incubation periods have the potential to become unproductive class time.

Here, we describe a semester-long CURE in which students manipulate DNA *in silico*, *in vitro*, and *in vivo* (see Appendix 1 in Text S1 in the supplemental material). To limit unproductive classroom time, we have developed stay-on-task exercises (SOTEs) (see Appendix 2 in Text S1). SOTEs are problems designed to be done during potentially unproductive incubation periods. The development of our CRISPR CURE and accompanying SOTEs provides students the benefit of course-based undergraduate research and offers instructors tools to maintain an effective learning environment.

## PROCEDURE

**FIG 1:**
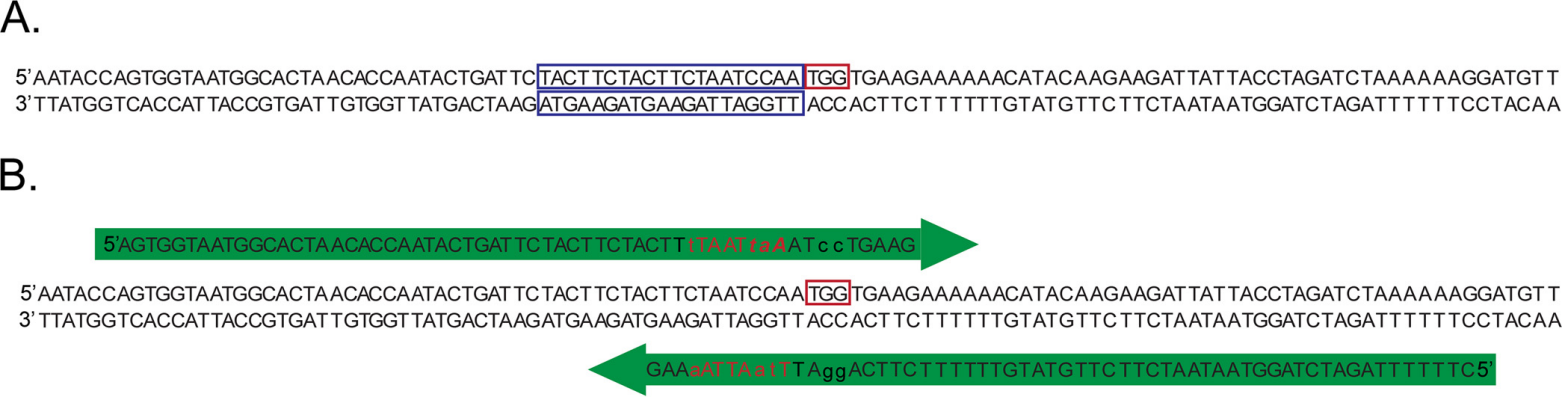
Cartoon of guide RNA and repair template design. A. Guide RNAs are outlined in blue and PAM is outlined in red. B. Repair templates are indicated in green. PAM sequence is outline in red. All changes in sequence are in lowercase. Stop codon that has been inserted is indicated by italics. The restriction site that has been inserted are red letters.

**FIG 2:**
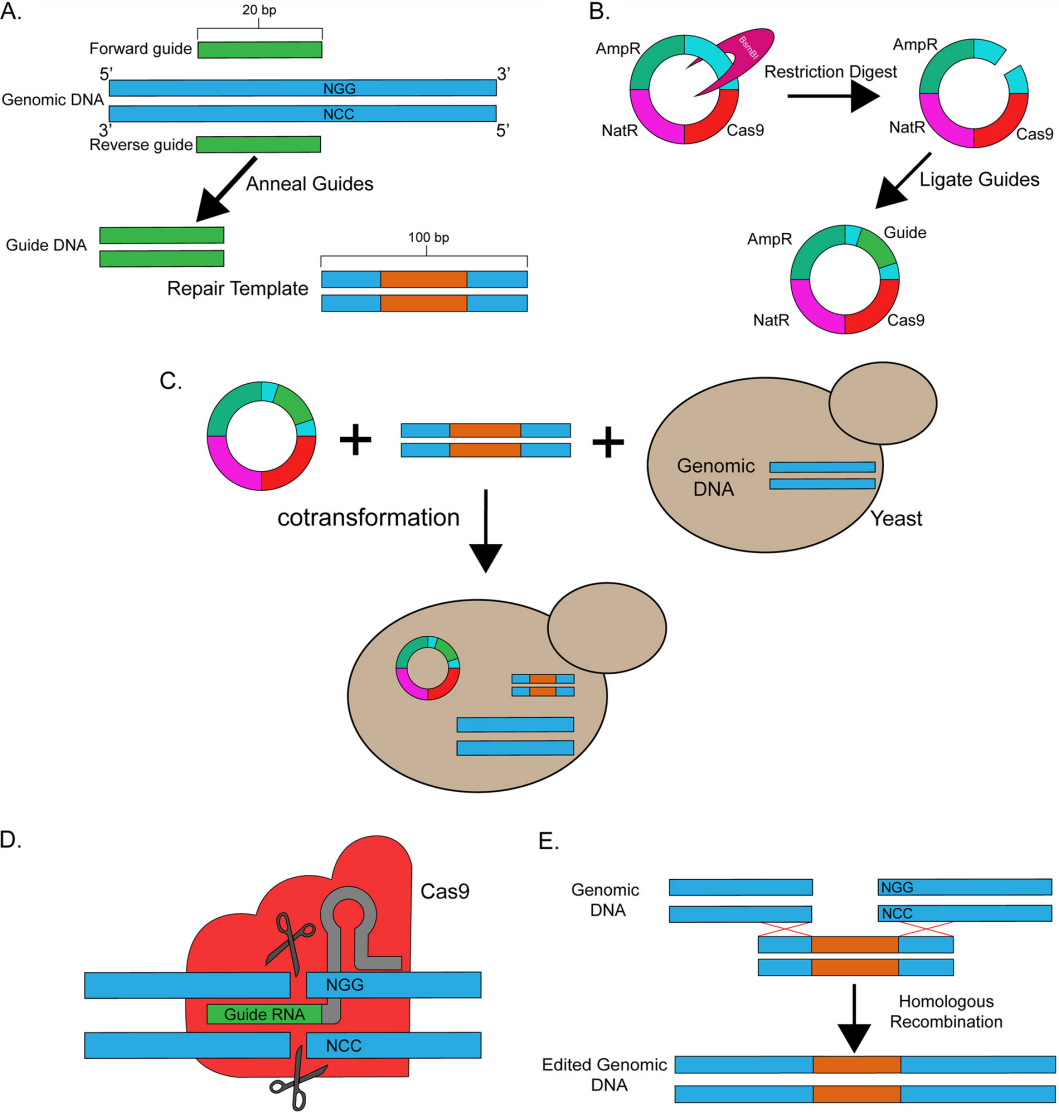
Cartoon of CRISPR workflow. Color schemes are consistent in A through E. A. Identification suitable guide sequences and repair templates. Guide sequence in green and repair templates in blue. Portion of repair template that will introduce the change into the genomic DNA is in orange. B. Guide RNA sequence is cloned into Cas9 expression vector. Cas9 gene is shown in red, cloning site is teal. C. Repair template and expression vector are transformed into yeast. D. Cas9 and guide RNA are expressed and cleave genomic DNA. E. DNA cleavage is fixed via homologous recombination with repair template.

Our CRISPR CURE is a one-semester course taken by undergraduates and graduate students with limited molecular biology experience. Students learn how to manipulate DNA *in silico*, *in vitro*, and *in vivo*. As our research lab is interested in RNA metabolism, we encourage students to choose genome edits that they hypothesize are important for these processes. The exercise as designed edits the Candida albicans genome, but similar CRISPR systems are available for many fungal species, and the principles outlined in Appendix 1 in Text S1 and in this article could be easily adapted to those systems. The first portion of the course requires students design guide RNAs and repair templates to edit the C. albicans genome. Guides and repair templates are developed by students using guidelines from the primary literature (5, 6). Students use Benchling software to design constructs; however, there are a variety of different software packages that could be used to perform oligonucleotide design (Fig. 1) (7, 8). Once students design guide RNAs, they clone them into a vector that expresses yeast-optimized Cas9 nuclease and encodes appropriate antibiotic resistance markers (Fig. 2) (5). Detailed procedures for these steps can be found in Appendix 1 in Text S1. All vectors that the students use are available from Addgene. Once guides have been successfully cloned into expression vectors and the sequences have been confirmed, these vectors along with repair templates are transformed into yeast. The repair templates introduce a mutation into a gene of interest and incorporate a restriction enzyme site subsequently used for screening transformants. Colonies are screened by colony PCR and restriction digestion to identify transformants containing the correct genome edit (9). Phenotypic analysis of the mutants can follow as time permits. All materials and instructions are provided in Appendix 1 in Text S1.

To combat some of the previously stated challenges posed by the asynchronous nature of the CURE class format and to further assess student learning, we have created SOTEs. These exercises, found in Appendix 2 in Text S1, challenge students to apply their knowledge of central dogma and molecular biology. Each problem is designed to be completed in less than half an hour. These problems can be assigned as a bolus early in the semester or can incrementally be assigned as the semester progresses. Students can work on these problems in teams or individually. We find that these problems provide an opportunity for stronger or more experienced students to teach their peers. The flexibility inherent in SOTEs allows students to work on them at their own pace. Depending upon the skill level and experience of the class, questions can be tailored so that they are challenging but not frustrating. Furthermore, as CUREs can vary in length, the number of questions can be adjusted so that students remain focused but are not overwhelmed by additional work. Finally, questions can be developed to complement a particular CURE exercise being performed. In our experience, SOTEs keep students focused on molecular biology during incubation periods and thus make for a more productive classroom.

## SAFETY ISSUES

The experiments described in this paper genetically modify C. albicans, a biosafety level 2 (BSL2) organism. In addition, plasmid must be purified for experiments from Escherichia coli. In the United States, the work described takes place at BSL2. Many undergraduate research labs are designed to work under BSL2 conditions. In cases in which it is not possible to abide by BSL2 guidelines, Saccharomyces cerevisiae can be substituted for C. albicans with only minimal changes to the protocol. If working in a different country, the laws guiding work with genetically modified organisms could differ and should be followed. Instructors and students should follow the biosafety guidelines of the American Society for Microbiology (ASM) (https://www.asm.org/Guideline/ASM-Guidelines-for-Biosafety-in-Teaching-Laborator).

## CONCLUSION

CUREs provide students with an opportunity to participate in innovative research projects in the organized confines of a classroom. Our CURE leverages CRISPR to manipulate the genome of the human fungal pathogen C. albicans. Each semester we have had students that have successfully edited the fungal genome. Even though not all students are able to successfully manipulate DNA *in vivo*, the students that do not make it to that stage have the opportunity to observe those that do and thus can vicariously experience these aspects of the CURE. At the end of this CURE, students can successfully explain the theory behind and practical applications of CRISPR-mediated genome editing techniques. These outcomes are measured by a variety of metrics, including exams, lab reports, and the SOTEs we have developed. SOTEs leverage the asynchronous nature of the CURE format to provide students with the opportunity to solve molecular biology problems related the CURE learning objectives. We find that the SOTEs provided in Appendix 2 in Text S1 complement the learning objectives for the CURE provided in Appendix 1.
