# Supplementary material for: Stay-on-Task Exercises as a Tool To Maintain Focus during a CRISPR CURE
Source: J Microbiol Biol Educ. 2021 Jun 30;22(2):e00114-21. doi: 10.1128/jmbe.00114-21 (PMC8442031; doi:10.1128/jmbe.00114-21)
Supplement: SUPPLEMENTAL FILE 1 — Download JMBE00114-21_Supp_1_seq1.docx, DOCX file, 9.9 MB [file jmbe00114-21_supp_1_seq1.docx]

**CRISPR Mediated Genome Editing of *C. albicans***

**Introduction:**

Genetic modification or mutagenesis is one tool scientists use to investigate organism phenotype. Genetic modification can be separated into two broad categories, random and directed. In random modification, a chemical or energy source damages or alters DNA randomly throughout the genome (1, 2). As these alterations are repaired changes to the DNA sequence are introduced. In directed mutagenesis damage is targeted to a specific DNA sequence of interest thus changes made during repair will be isolated to the targeted DNA sequence. This semester we will perform targeted mutagenesis of a *C. albicans* gene (3). The genes chosen as potential targets are proposed to play roles in RNA metabolism but their functions have not been confirmed (4).

One way to perform targeted mutagenesis is through genome editing using Clustered Regularly Interspaced Short Palindromic Repeats or (CRISPR). CRISPR is an efficient method of performing targeted mutagenesis(5, 6). CRISPR was discovered in bacteria where it cuts invading viral DNA in a sequence specific manner acting as a bacterial immune system (7, 8). Scientists are leveraging the power of CRISPR to edit the genomes of many organisms. The application of this technology is changing the landscape of both industrial and academic science. Three things are required to edit a genome with CRISPR **1)** Cas9 a nuclease that will cut the genome **2)** A guide RNA, which will guide Cas9 to the specific sequence of the genome using base pairing **3)** Repair template which will repair the double stranded break introduced by Cas9 and in doing so introduce a mutation to the genome (9).


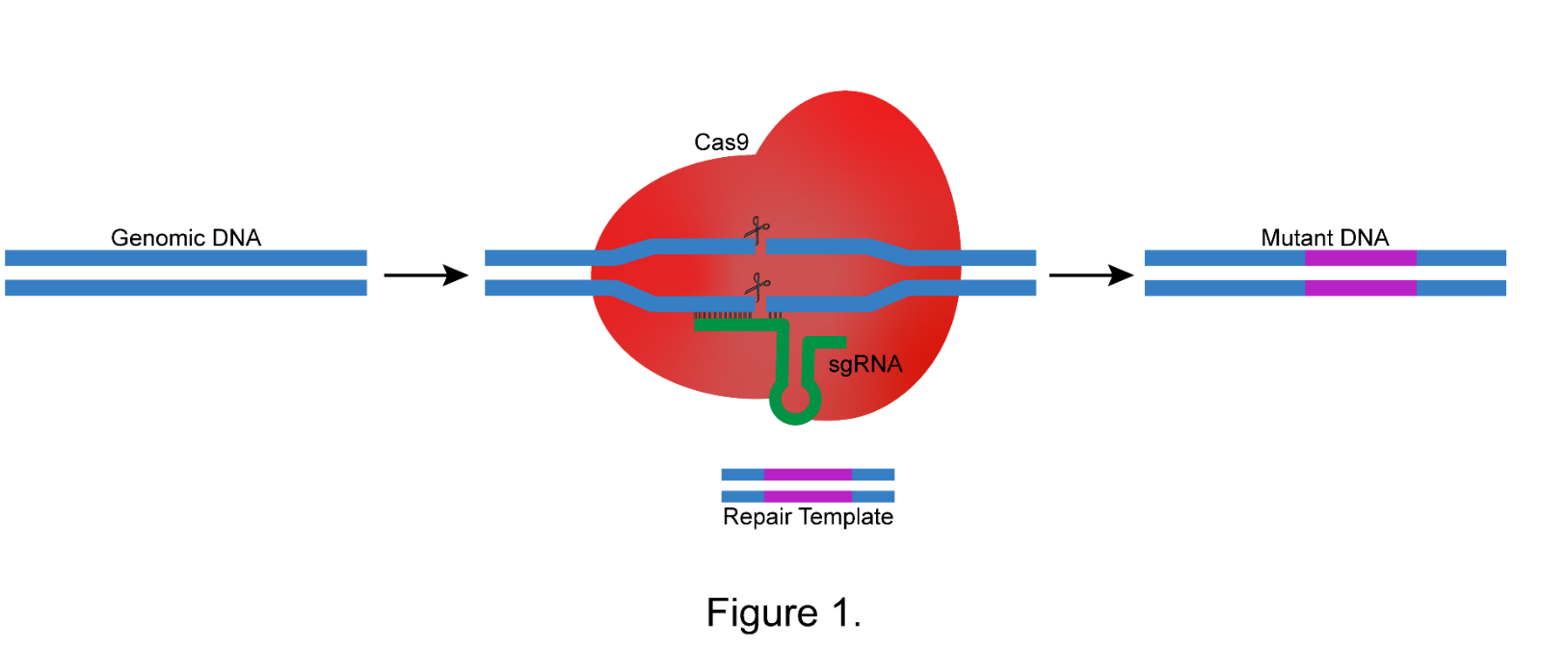
**Figure 1**

A plasmid encoding a yeast optimized version of Cas9 is provided (10). Throughout the semester, your goal will be to clone a guide RNA sequence into this plasmid. In addition, you will make a repair template by primer extension. Simultaneous, transformation with both the repair template as well as the cloned plasmid will introduce all of the necessary genome editing components to the yeast cell and will facilitate genome editing. You will then screen your transformants to identify those that harbor the correct mutation.

**Methods:**

To edit a genome with CRISPR we need 3 things; Cas9 a nuclease that will cut the genome, a guide RNA, which will guide Cas9 to the specific sequence of the genome using base pairing, and the repair template which will repair the double stranded break introduced by Cas9 and in doing so introduce a mutation to the genome. One popular way to introduce these components is by encoding them on a plasmid. You will clone your guide RNA sequence into a plasmid that encodes Cas9 optimized for expression in yeast. When this vector is transformed into a yeast cell it will express Cas9 and guide RNA. The guide RNA will bind to Cas9 and target it to complementary sequence in the yeast genome.

The steps laid out below can be performed in as few as 2 days or can be spread out over a series of weeks depending upon the time available. Resources for guide sequences for *Candida* species are available at (11)

**Identification of a suitable guide sequence:**

There are **4** rules that must be followed when picking a guide sequence. **1)** The guide sequence must be directly 5’ of a PAM site (NGG) where N is any nucleotide. Cas9 requires the PAM site to cut so if your guide is not downstream of one even Cas9 will not cleave the DNA. **2)** The guide sequence cannot contain more than 5 Ts in a row as the polymerase used to express the guide works poorly on polyT tracts of DNA. **3)** The guide must be 20 bases long **4)** At the ends of the guide sequences, you must add sequences that allow for cloning into the cut plasmid. These additional residues are not part of the 20 nucleotides. Examples of these sticky ends are shown at the bottom of this page. You can identify guide sequences to your gene of interest in vector NTI or a similar computer program. Alternatively you can do it by hand. Examples of guide sequences are shown below, Figure 2, 3, and 4.

**Figure 2**


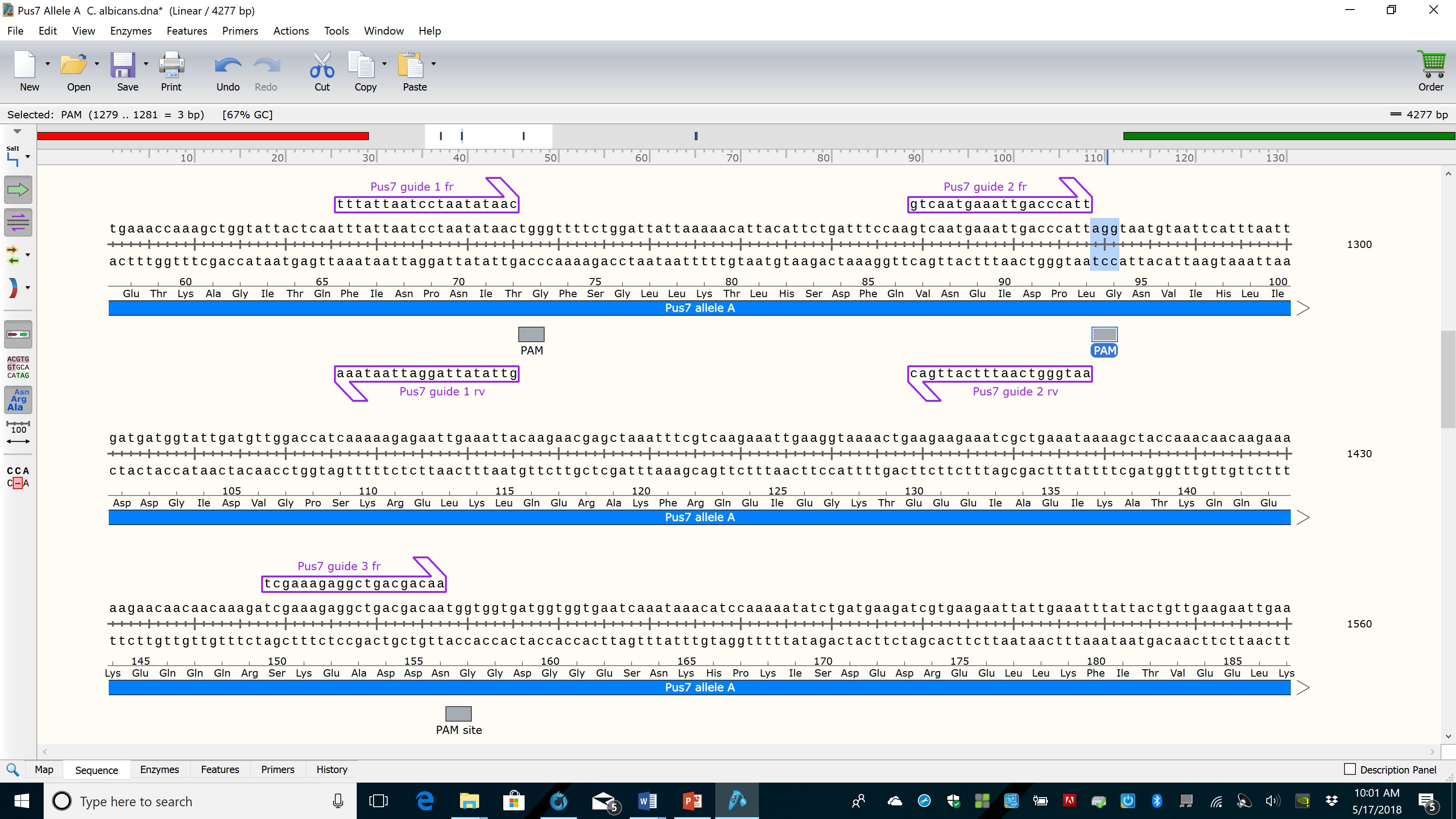


Example of primers that you would make if you wanted to clone Pus7guide 1 fr and rv

Sticky Ends for cloning are indicated by lowercase letters, these are used for cloning into pV1382, pV1393, pV1524, or pV1093 and are added during guide design.

Pus7DAB1fr 5atttgTTTATTAATCCTAATATAACg

Pus7DAB1rv 5aaaacGTTATATTAGGATTAATAAAc


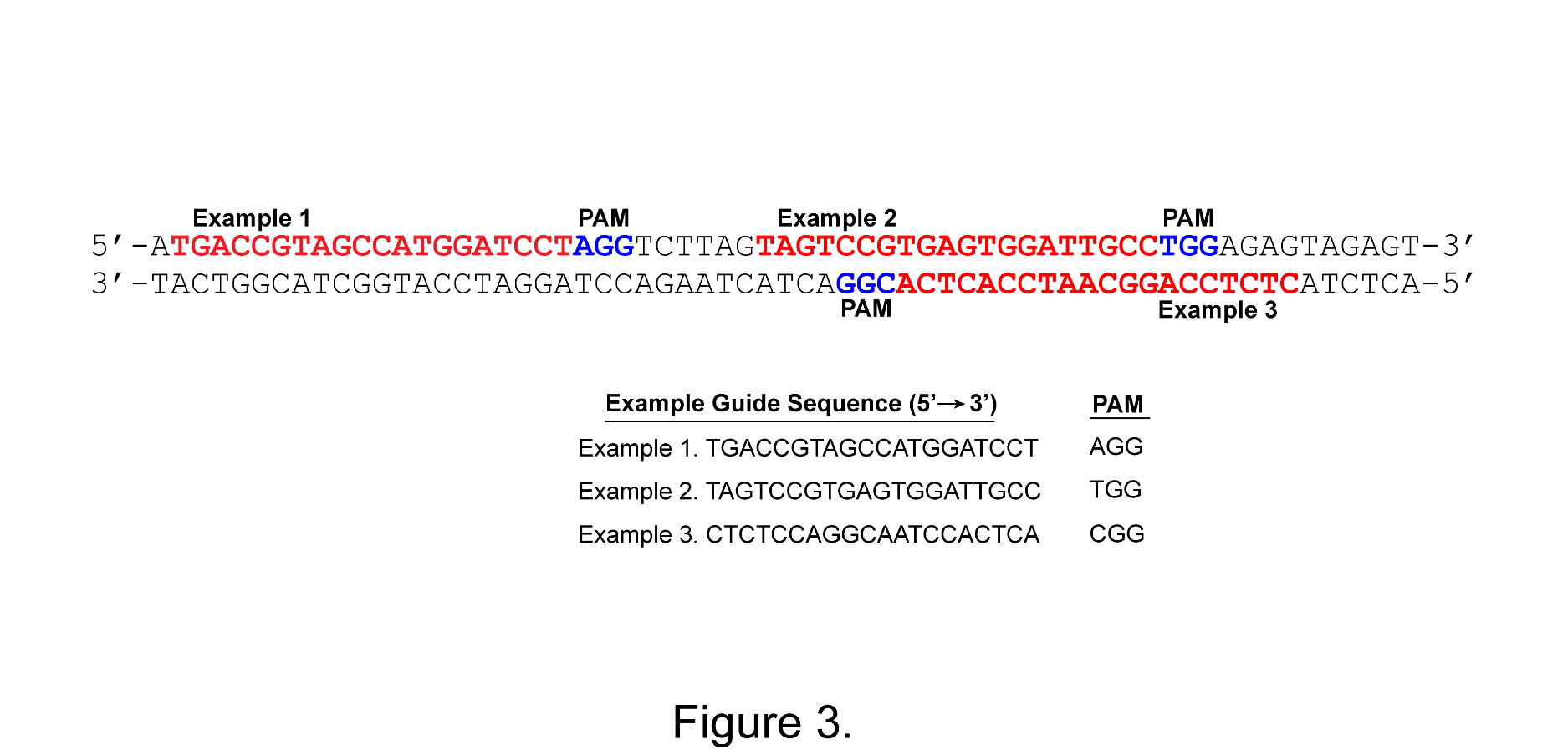
**Figure 3**

More examples of PAM sequences can be found in Figure 2. Each group of two students must generate 4 sets of guides close to the 5’ end of their assigned gene. You must provide these sequences to your instructor in an excel file in the following format.

Column 1 Name of Primer (GeneNameyourinitials#fr) No spaces The reverse primer will end with rv not fr.

Column 2 Primer Sequence **(GUIDE SEQUNECE CAPITALIZED) (sticky ends lower case)**

You should save the file that contains your guide sequences and print the file for your notebook.

**Clone guides:**

1. **Cut plasmid expression vector (pV1382, pV1393, pV1524, or pV1093) with BsmBI:**
   1. Prepare a heat block or water bath at 55°C.
   2. Digest 2 μg of DNA by adding the following to a 1.5 ml tube
      1. DNA 2 μg
      2. 10x NEB3.1 5 μl
      3. BsmBI 1 μl
      4. H_2_O to 50 μl
   3. Incubate at 55°C for 20 min.
   4. Cool to room temperature and spin to bring down any condensation from the top of the tube.
   5. Add 1 μl Calf Intestinal Phosphatase
   6. Incubate at 37 °C for 1 h.
   7. Purify using PCR Purification Kit (Instructions provided with kit); elute in 30 μl EB.
2. **Phosphorylate and Anneal GuideRNA oligos**
   1. Add to a PCR tube:
      1. 100μM Oligo 1 (top) 0.5 μl
      2. 100μM Oligo 2 (bottom) 0.5 μl
      3. 10x T4 Ligase Buffer 5 μl
      4. T4 Polynucleotide Kinase 1 μl
      5. H_2_O 43 μl
      6. (also make a negative control with no oligos)
   2. Incubate in a thermocycler:

a. 37°C for 30 min

b. 95°C for 5 min

c. Cool to 16°C, at the slowest ramp rate your machine can do

d. Alternatively this can be done on a heat block if you use a 1.5 ml tube.

1. **Ligate Annealed Oligos**
   1. Assemble in a PCR tube:
      1. 10x T4 Ligase Buffer 1 μl
      2. Ligase 0.5 μl
      3. Annealed Oligo mix 0.5 μl (include a negative control w/no oligos)
      4. Vector 20-40 ng
      5. H_2_O up to 10 μl final volume
   2. Incubate in a thermocycler:
      1. 16°C for 30 min
      2. 65°C for 10 min
      3. cool to 25°C
2. **Transform ligation into chemically competent DH5α**
   1. Mix 50 μl competent cells and 5 μl ligation product or negative control in a labeled 1.5 ml tube.
   2. Close both tubes and set both tubes on ice for 10 min.
   3. Heat shock tubes of cells by putting them in the 42°C water bath for 45 sec.
   4. Remove the tube from the bath and place on ice for 3 min.
   5. Add 200 μl LB liquid media
   6. Place at 37°C for 30 min.
   7. Pipette ligation onto the amp agar plate **(label plate)**
   8. Pipette negative control onto second Amp agar plate **(label plate)**
   9. Pour 10-20 glass beads onto each plate and put the lid back on each plate. Shake the plates for 20 sec. Pour the glass beads into the dirty bead container. Alternatively, one can spread the cells using a cell spreader.
   10. Place plates upside down in 37°C incubator overnight.
   11. After 24 h take plates out of the incubator.

**5.** **Confirm ligations by sequencing**

- 1. Start 5 ml LB overnight cultures with 5 ul Amp. Sequencing 4 clones is typically sufficient to identify multiple correct insertions.
  2. Miniprep plasmid DNA (Instructions provided with kit.)
  3. Sequence the insertion by adding 2 μl plasmid miniprepped DNA, 0.25 μl primer, and 12.75 μl H_2_O to a PCR tube. Submit your tubes for Sanger sequencing with either a core or commercial facility.
  4. The primer we use is Snr52-fwd- 5’GGCATAGCTGAAACTTCGGCCC3’. This corresponds to the promotor region upstream of where the guide will be insterted.
  5. Analyze sequences to identify clones that contain your guide sequences. Correct plasmids can now be used for CRISPR mediated mutagenesis of *C. albicans*.

**Repair Template Design:**

The sequence of the repair template used will introduce the intended mutation. In this exercise, you will introduce a stop codon to effectively knockout the targeted gene. These mutations have the benefit of being able to be screened through restriction digestion as opposed to sequencing saving time and money. If a different type of mutant is desired, DNA sequencing of PCR products will be required to identify correctly edited genomes. A table of restriction sites that when introduced code for either stop codons or amino acid changes is listed below and in (12).

**Table 1**

**Restriction sites that encode consecutive amino acids or stop codons.**

| **Translation Product** | **Codon** | **Restriction Enzyme Recognition Site Sequence** |
| --- | --- | --- |
| **2xAlanine** | GCN | **BbvI** GCAGCN (8/12), **Fnu4HI** GC/NGCN, **TseI** G/CWGCN |
| **2xArginine** | CGN | **Hpy99I** CGWCGN/ |
| **2xGlutamate** | GAR | **BseRI** GAGGAG (10/8) |
| **2xGlycine** | GGC+GGA | **EciI** GGCGGA (11/9) |
| **1x Stop Codon** | TAG | **SpeI** A/C*TAG*T |
| **1x Stop Codon** | TAA | **PacI** T*TAA*T/TAA |
| **1x Stop Codon** | TGA | **AcuI** C*TGA*AG(16/14) |

**Table Legend:** Restriction enzyme recognition sites that encode consecutive identical amino acids or stop codons.  **W** = A or T, **M** = A or C, **K** = G or T, **R** = A or G, **N** = A T C or G, **/**=cleavage site. Numbers in parentheses correspond to how far upstream a cut site will occur from the recognition sequence.

Repair templates will be designed using DNA editing software. I recommend benchling, although there are other free software that can be used.

**1)** Make sure to save the repair template file as a separate file as you will be making changes to your DNA sequence. Make a new file for each new set of repair templates.

**2)** Identify where your guide sequence will target. You want the mutations you introduce to be close to the cleavage/guide site. Deleting the PAM site or mutating the guide region will make it so that the region cannot be further targeted by Cas9.

**3)** Introduce the restriction site to the gene making sure that the stop codon encoded by the site is in frame. If you are not in frame the stop codon will not be read and thus translation will not terminate.

**4)** Design repair templates for all successfully cloned guides by making two 60 base oligonucleotides that have 20 bases of complementary sequence on their 3’ ends.

See Figures 4 and 5.

You must provide these sequences to your instructor in an excel file in the following format.

Column 1 Name of Primer (GeneNameyourinitials#reptempfr) No spaces

Column 2 Primer Sequence Mutations you have made CAPITALIZED

Save files that contain your repair sequences and print the file for your notebook.

Example provided on the next 2 pages (Figure 4 and 5).

Bases that have been inserted are capitalized and red, the red asterisk indicates a stop codon.

**Figure 4**


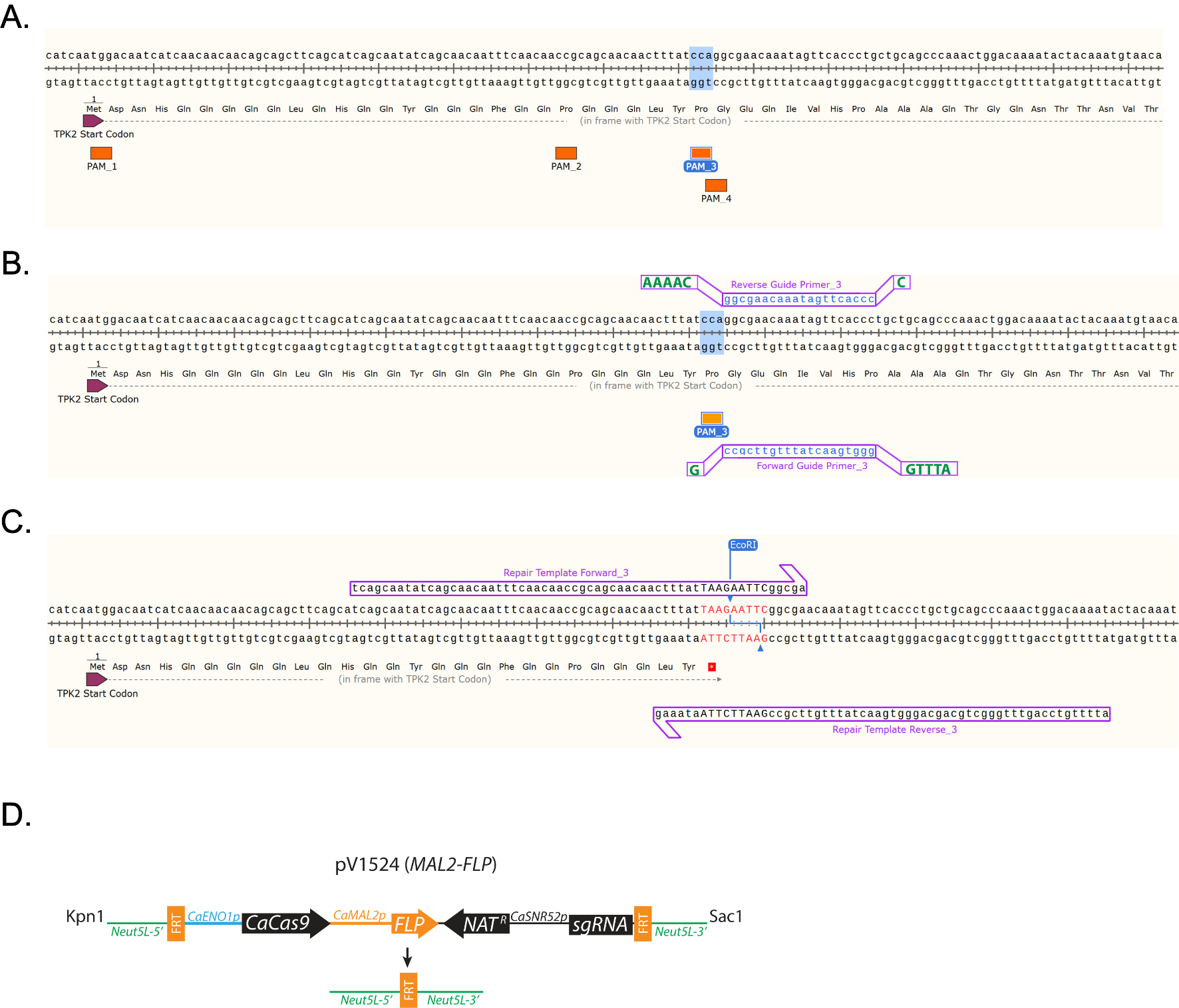


**Generation of Repair Template:**


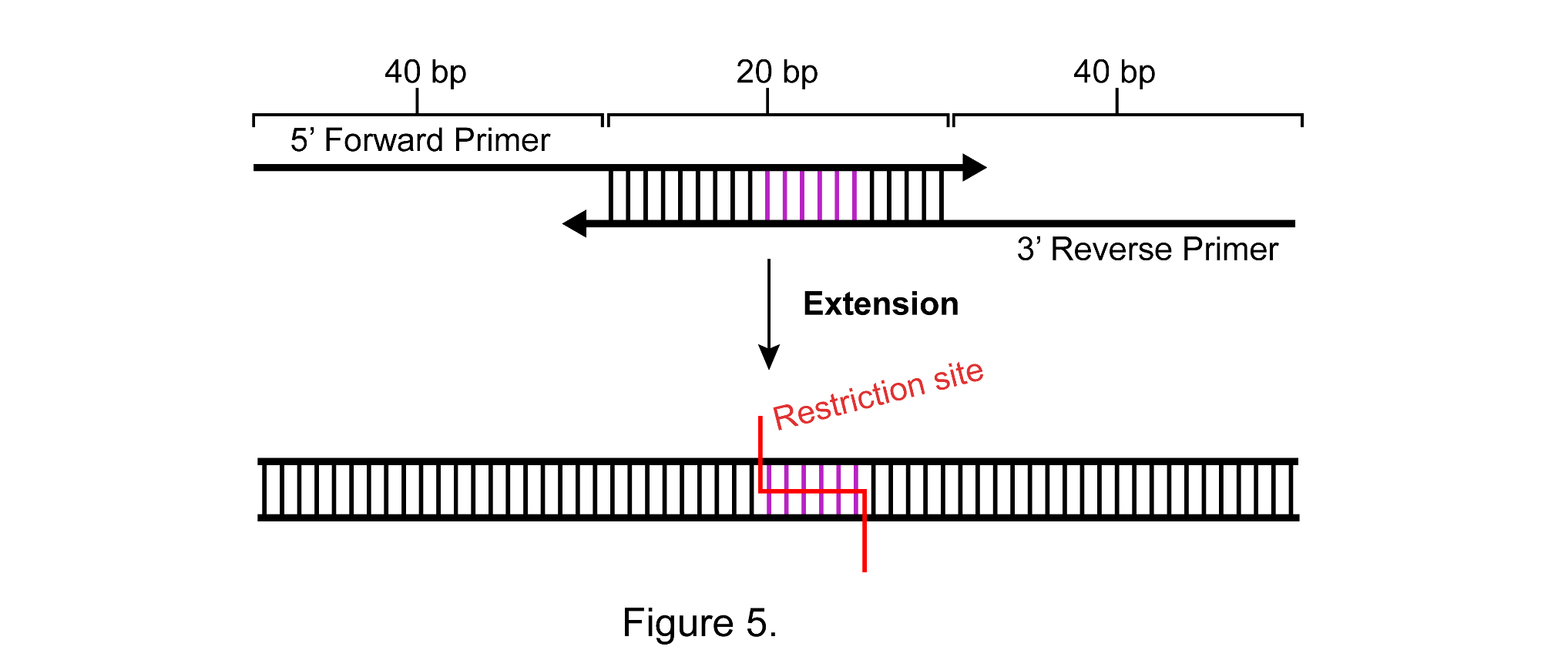
**Figure 5**

We will create repair template using 60bp oligos with ~20bp overlap at 3’ end shown below, we will subject them to PCR (4x60µl reactions, which are then pooled and purified) generating a product of ~100bp. These products will then purified using a QIAquick PCR purification column or similar PCR purification protocol, and eluted in 50 µl; typical final concentration of the primer extension product is ~200-300 ng/µl.

**To each of four PCR tubes add**

1. 1.2 μl 100μm Fr repair template primer
2. 1.2 μl 100μm Rv repair template primer
3. 6 μl dNTPs
4. 6 μl Buffer
5. 0.6 μl Taq polymerase
6. 45 μl H2O

One master mix can be made and divided into four tubes to save time.

**Example Primer Extension Conditions**

Step 1 95°C 2 min

Step 2 95°C 20 sec

Step 3 52°C 2 min

Step 4 68°C 1 min

Repeat Step 2-4 30X

Step 5 68°C 5 min

Step 6 4°C ∞

**Purification of Repair Template**

We now need to purify our repair template. This step will concentrate the DNA and get rid of the salt from the PCR. Instructions below use qiagen components but other commercially available and homemade PCR kits would work just as well.

1. Add 5 volumes of buffer PB1 to your PCRs

2. Place a spin column in a 2 ml collection tube

3. Centrifuge for 30-60 sec at maximum speed

4. Discard flow through and put column back in collection tube, your DNA is now bound to your column.

5. Wash DNA with 750 μl Buffer PE (wash buffer), (make sure that ethanol has been added to the PE or you will lose your sample.)

6. Centrifuge for 30-60 sec at maximum speed

7. Discard flow through and put column back in collection tube

8. Centrifuge for 1 min at maximum speed to get rid of residual wash buffer

9. Elute in 50 μl of EB (elution buffer) by centrifuging for 1 min at maximum speed

An overview of guide cloning and repair template development can be found in (13)

**Digestion of plasmids to prepare DNA for transformation**

Cut plasmid with Kpn1 and Sac1 to release the fragment that will insert at *ENO1* locus upon transformation into *C. albicans*. ***Make sure to keep all enzymes on ice during laboratory experiment set up!***

Add the components listed below to a 1.5 ml tube in the order listed and incubate at 37°C overnight.

1. 14.6 μl sterile H_2_O
2. 4 μl 10X Cut Smart Buffer
3. 0.4 μl BSA (10mg/ml)
4. 20 μl miniprep of plasmid pv1093 that contains your guide
5. 0.5 μl Kpn1 HF
6. 0.5 μl Sac1 HF

***C. albicans* transformation**

**Materials for the *C. albicans* transformation:**

- **PLATE**

40% PEG 3350

100 mM Lithium Acetate pH 7.5

10 mM Tris-Cl pH 7.5

1 mM EDTA pH 7.5

- **Salmon Sperm DNA**, boiled for 5-10 min then placed on ice
- **Transforming DNA** which is the cut plasmid and repair product. Greater than 10 μg is optimal, but since we are under a time constraints we will use whatever we have.
- **TE/LiOAc**

10 mM Tris-Cl pH 7.5

1 mM EDTA pH 7.5

100 mM Lithium Acetate pH 7.5

**Protocol for *C. albicans* transformation**

**1.** Grow overnight YPD+Uri culture of *C. albicans* at room temperature (started Day 1), ideally to OD_600_ less than 6. (This should be one of your overnight cultures from the previous page)

**2.** Spin down 5 OD_600_ units of cells per transformation. Spin 5 minutes at 5000 RPM in benchtop centrifuge. Discard the supernatant.

1 ml of culture with an OD_600_ of 5 is 5 OD_600_ units; this is enough for one transformation. If your OD_600_ is 2.5, you will need 2 ml for 5 OD_600_ units.

**(Make sure to prepare an additional transformation in a separate 1.5 ml tube in which you do not add any DNA. This will serve as a negative control, as you should not get any transformants!)**

**3.** Suspend the 5 OD_600_ units of pelleted cells in 100 μl TE/LiOAc.

**4.** To an Eppendorf tube add the following in the order listed.

1. 100 μl cells
2. 40 μl salmon sperm
3. Ideally more than 10 μg transforming DNA (plasmid digestion)
4. 1 ml PLATE

**5.** Mix gently by pipetting. Let sit over night at room temperature.

**Day 3**

**1.** Heat Shock at 44°C for 25 min.

**2.** Spin 5 min at 5000 RPM in a benchtop microcentrifuge

**3.** Remove PLATE mixture and wash once by adding 1 ml of YPD and centrifuging again for 5 min at 5000 RPM.

**4.** Suspend in 0.1mL YPD and incubate on a roller drum or shaker at room temperature overnight

**Day 4**

**1.** Plate on YPD plates with 200 μg/ml nourseothricin (2X Nat) media

**2.** Colonies will appear in ~3 days

**3.** Count the number of colonies on each group’s plates.

| **Table** | **Strain** | **Number of Colonies** |
| --- | --- | --- |
| **Group 1** |  |  |
| **Group 2** |  |  |
| **Group 3** |  |  |
| **Group 4** |  |  |

**Day 5 Streak for Single Colonies**

When you plate your transformation you will likely be plating hundreds of thousands of live cells. Some of these cells will have been transformed and will express the antibiotic resistance we intended. Some will harbor the resistance gene but will not express it well and will thus grow slowly. Some of the cells will have undergone alternative mutation events that allow them to grow on antibiotic, but won’t harbor the resistance genes. All of these cells will grow into colonies next to one another and may become indistinguishable from one another. For all down-stream applications it will be important that we work with a pure culture, and as such we need to separate individual colonies. We do this by streaking for single colonies. The goal of this is to isolate single cells that can grow into a colony and thus the members of the colony will be genetically identical.

For our purposes you will be streaking 8 colonies (4 colonies per plate).

**Figure 6**


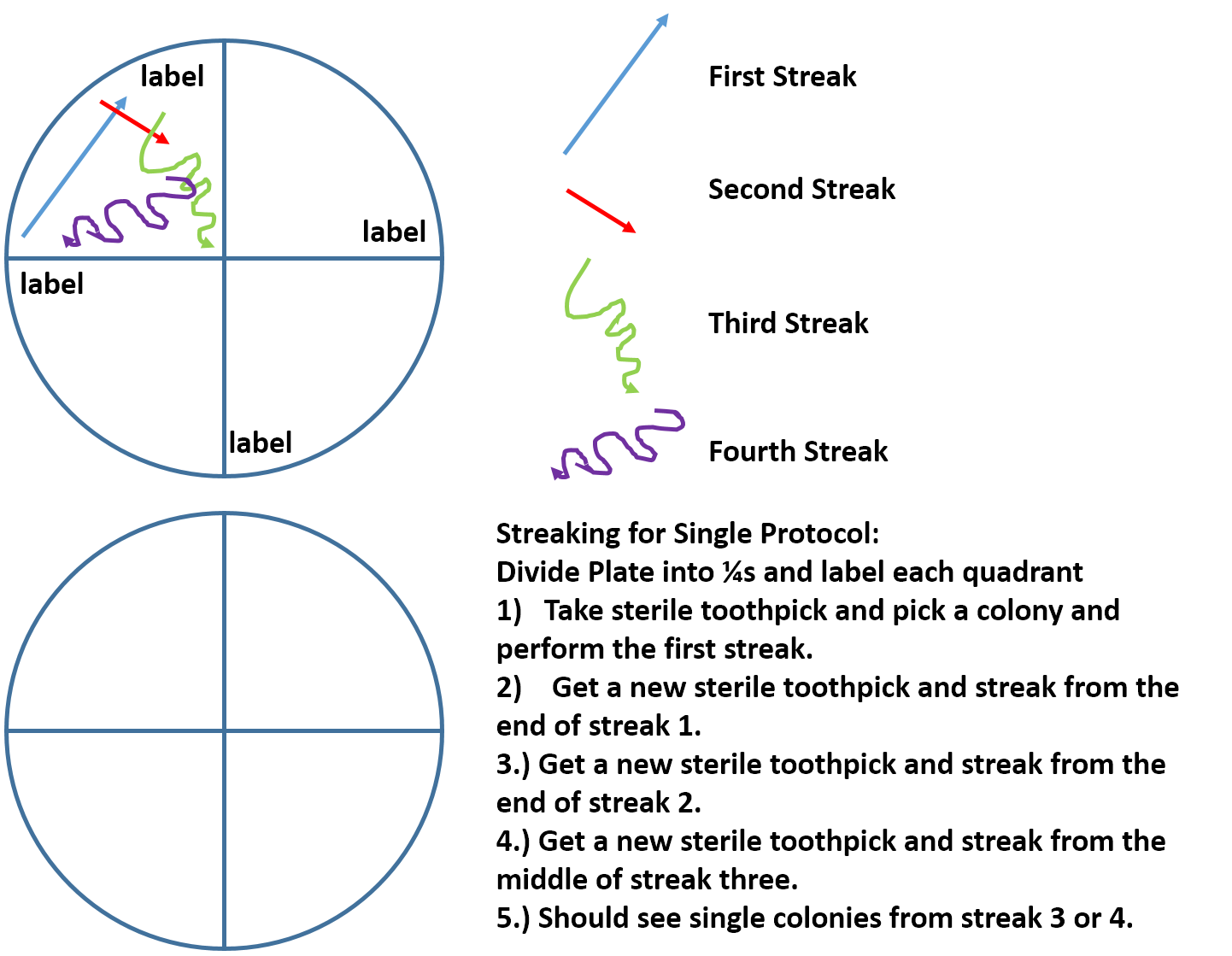


Always streak onto a plate with antibiotic so that cells that have not been transformed are unable to grow. Single colonies will need to grow for at least 3 days.

**Design Primers to Verify Transformants**

We now need to see if genome editing was successful. We will amplify the regions that flank the mutated region by PCR and subject the PCR product to digestion with a restriction enzyme whose site was introduced during the mutagenesis. To do this we must perform colony PCR. Colony PCR is similar to PCR from a plasmid or piece of exogenous DNA, but instead you are using whole cells (that contain your DNA) as your template DNA. Primers should be designed such that a 500 base fragment is amplified with the desired mutation 200 bases from one of the primers. See diagram below. Primers can be designed with DNA editing program that you used to design your other primers.

**Figure 7**


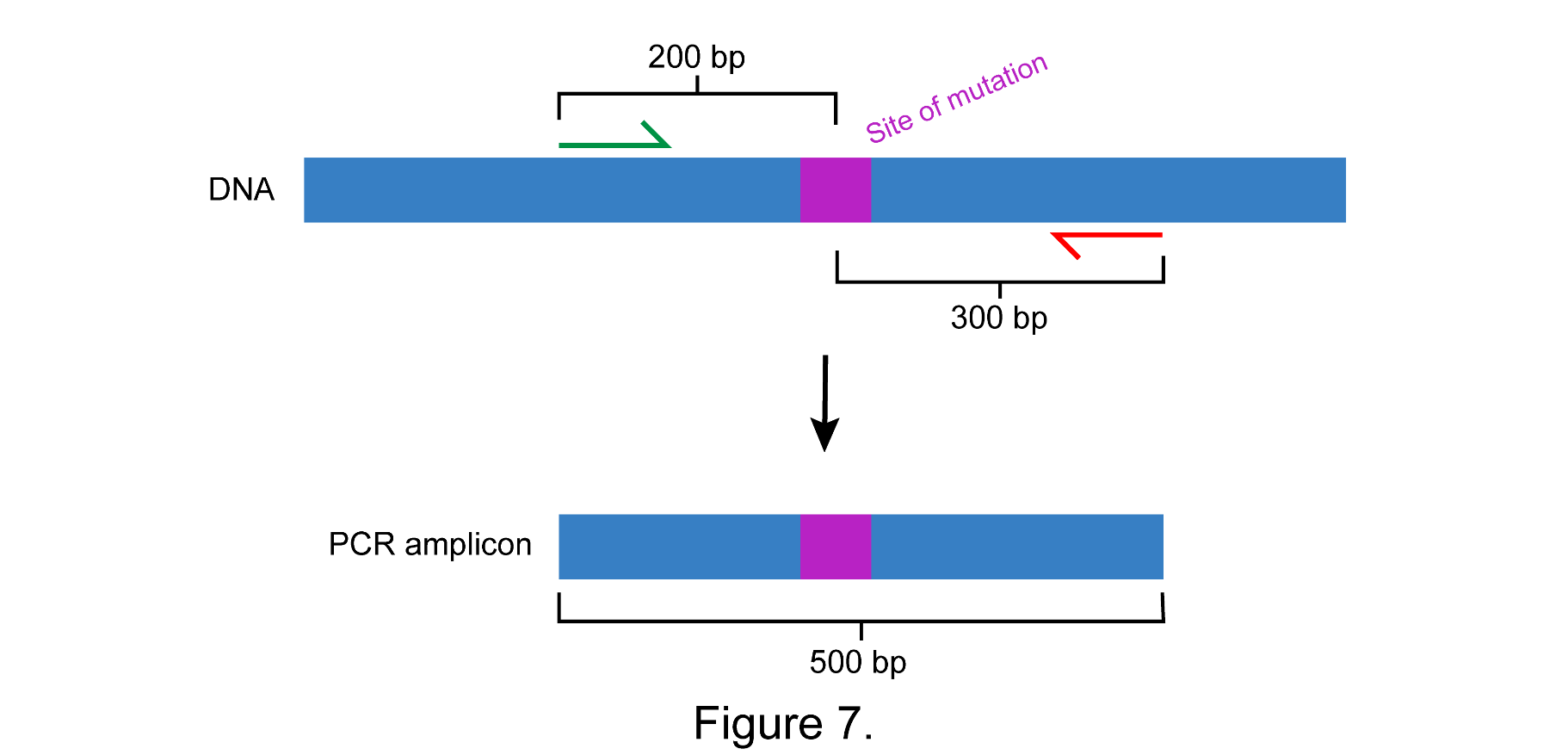


Design 2 sets of Check Primers for all successful transformations. Primers must be between 20 and 30 bases long and of suitable melting temperatures to perform PCR. There are many programs available online to assist you in designing suitable primers. Depending upon where your guide targets you may need to design primers upstream from your gene of interest. This sequence information can be found in the *Candida* genome database.

You must provide these primer sequences to me in an excel file in the following format.

Column 1 Name of Primer (GeneNameyourinitials#chprifr) No spaces

Column 2 Primer Sequence

Save the files that contains your check primer sequences and print the file for your notebook.

**Verify transformants**
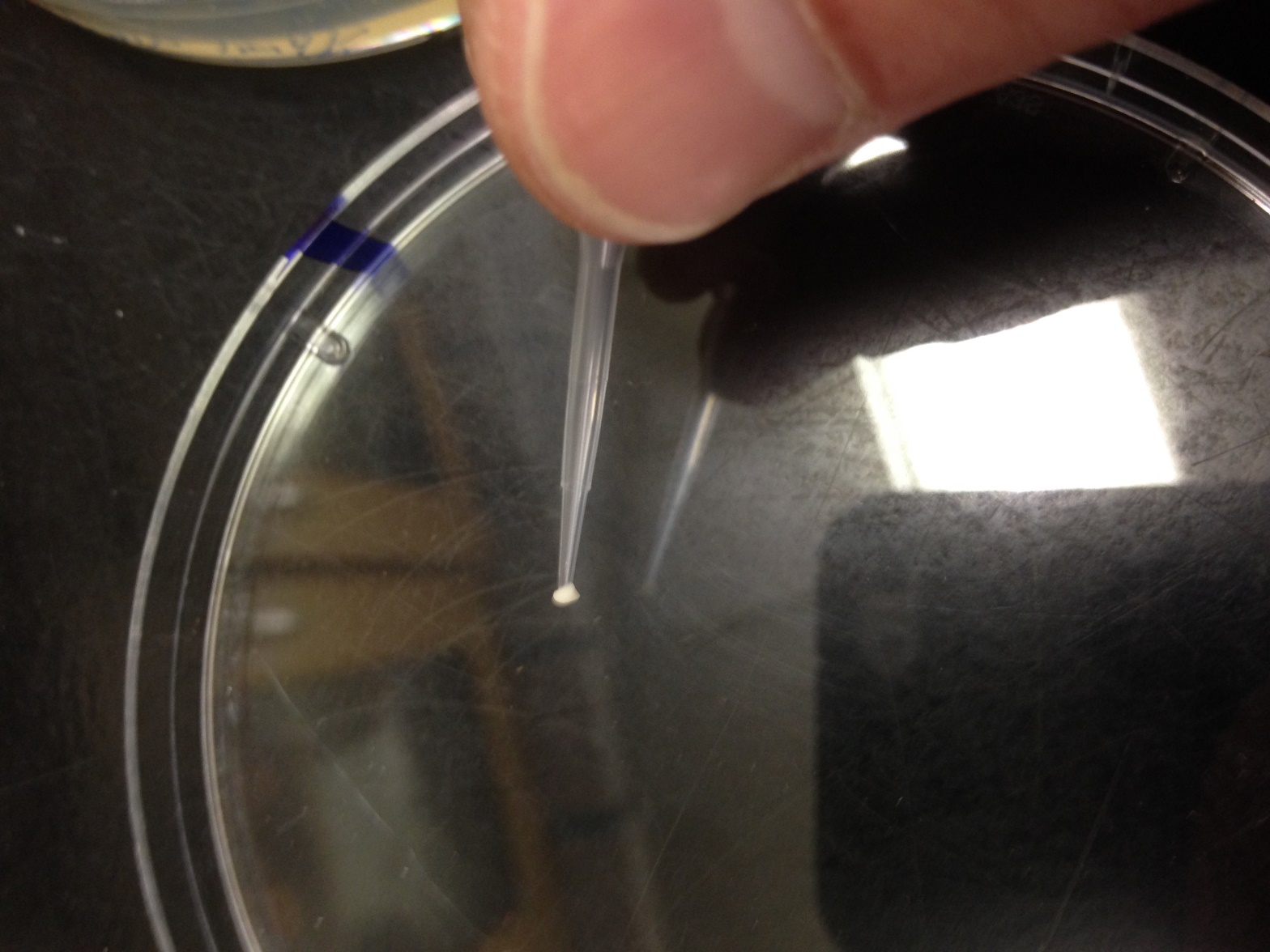


**Colony PCR protocol**

**1)** Add below components to a 1.5 ml tube: **Figure 8**

1. Check Primer Fr 0.3 μl x 9 = 2.7 μl
2. Check Primer Rv 0.3 μl x 9 = 2.7 μl
3. Thermostable Polymerase (ExTaq) 0.3μl x 9 = 2.7 μl
4. dNTPs 3 μl x 9 = 27 μl
5. ExTaq Buffer 3 μl x 9 = 27 μl
6. H_2_O 23 μl x 9 = 207 μl

**2)** Aliquot 30 μl into each of eight PCR tubes.

**3)** 0.25 μl of single yeast colony to each tube, approximately the amount of yeast shown on the right at the end of the P10 tip, red arrow is pointing at the yeast.

***Note:*** *Everyone will test 8 colonies provided we have enough colonies. It is wise to make up a master mix of all components with the exception of the colony which will be added separately to each tube after the master mix has been added. I usually make a master mix that is slightly more than I will need. This way it allows for liquid that gets stuck the tip or in a tube. Generally adding 10% is sufficient, thus why I multiplied everything by 9.*

**4)** Run colony PCR program to amplify the DNA and resolve on an agarose gel.

(Adapted protocol for gel electrophoresis from Addgene can be found on the next page.)

**Gel Electrophoresis (Adapted from Addgene.org)**

**Pouring a 1% Agarose Gel**

1. Decide on the volume of gel you wish to make.

Measure out 1 g of agarose for every 100 ml of gel you would like to pour.

1. Pour agarose powder into microwavable flask along with 1xTAE (TRIS-Acetate-EDTA buffer). TAE recipe available online.
2. Microwave for 1-3 min (until the agarose is completely dissolved and there is a nice rolling boil). Usually ~one minute per 100 ml of gel.

***Note:*** ***CAUTION*** *this liquid is very hot! Be careful stirring, as eruptive boiling can occur.*

1. Let agarose solution cool for 5 min.
2. Add ethidium bromide (EtBr) to a final concentration of approximately 0.2-0.5 μg/mL. EtBr binds to the DNA and allows you to visualize the DNA under ultraviolet (UV) light.

***Note:*** ***CAUTION*** *EtBr is a known mutagen. Wear a lab coat, eye protection and gloves when working with this chemical.*

1. Pour the agarose into a gel tray with the well comb in place. (Bubbles are not your friend and they can be eliminated with a pipette tip or by spaying a fine mist of ethanol solution over the gel.)
2. Place newly poured gel at 4°C for 10-15 min OR let sit at room temperature for 20-30 min, until it has completely solidified.

**Loading Samples and Running an Agarose Gel:**

1. Add loading buffer to each of your digest samples. The loading buffer that we are going to use is 6X. We will run 12 μl of our digestion so we will need 3 μl of loading buffer per sample.

***Note:*** *We add loading buffer for two reasons: 1) It provides a visible band that helps with gel loading and also allows you to gauge how far the gel has run. 2) It contains glycerol, so that, after adding it, your sample is heavier than water and will settle to the bottom of the gel well, instead of diffusing in the buffer.*

1. Once solidified, place the agarose gel into the gel box.
2. Fill gel box with 1xTAE until the gel is covered. Our large gel box will require roughly 2 L.

***Note:*** *Since the gel we will be running is very large and we added EtBr for visualization, we must add EtBr to the buffer as well. EtBr is positively charged and will run in the opposite direction from the DNA. If you run the gel without EtBr in the buffer, you will reach a point where the DNA will be in the bottom portion of the gel, but all of the EtBr will be in the top portion. In this case, bands in the top and bottom portions of the gel will be differentially intense.*

1. Load a molecular weight ladder into the first lane of the gel and once in the middle of the gel.
2. Carefully load your samples into the additional wells of the gel.

***Note:*** *Use two hands to load your gel. I usually guide with my left hand and pipette with my right, but you can reverse if you are lefty. To test if you are in the well you can press the pipette tip lightly against the side of the well. If you are not in the gel you will not feel the gel wall. Do not press too hard as this will break the gel.*

1. Run the gel at 200 V until the dye line is approximately 70% of the way to the next set of wells.

***Note:*** *The black electrode is negative, and the red electrode is positive. The DNA is negatively charged and will run towards the positive electrode. A useful mnemonic device is to remember to* ***always Run to Red.***

1. Turn OFF power, disconnect the electrodes from the power source, and then carefully remove the gel from the gel box.
2. We will visualize our gel on the gel doc at the end of the hall.

***Note:*** *When using UV light, protect your skin by wearing safety goggles or a face shield, gloves and a lab coat.*

**Analyzing Your Gel:**

Using the DNA ladder in the first lane as a guide (the manufacturer's instruction will tell you the size of each band), you can interpret the bands that you get in your sample lanes to determine if the resulting DNA bands that you see are as expected or not. Take a picture of the gel and save in your notebook. If you see bands of the proper size proceed to digestion.

**Restriction digestion of colony PCR products**

Add the following contents to a 1.5 ml or PCR tube.

1. 10 μl PCR product (Making sure not to disturb the cell pellet at the bottom of the tube)
2. 3 μl Appropriate 10X buffer
3. 1 μl Restriction Enzyme (Make sure to keep the enzyme on ice.)
4. 16 μl H_2_O

Incubate according to manufacturer’s instructions.

After digestion run gel as above and take a picture of the gel.

**Saving Correct Yeast Strains**

When one edits the genome of an organism the ultimate goal is to see how those edits effect phenotype. To do this one must save strains that have been correctly edited so that they can be used later in these experiments. Furthermore reagents used to make the strains such as plasmids that encode the guide RNA also need to be saved. The easiest way to save bacterial and yeast colonies is by freezing them at -80°C. Frozen stocks can be saved for many years.

Label 2 ml tubes capable of being stored in the -80°C on the top and side with the strain name. To save a strain, add 1 ml of an overnight culture to a tube. To this, add 1 ml of 40% glycerol. This will make a final volume of 20% glycerol. Mix the solution by inverting the tube five or six times. The glycerol will allow the yeast or bacteria cells to freeze but limit ice crystal formation which can kill cells. This should be done for both correctly edited yeast strains as well as the bacteria containing the plasmids with guide sequences cloned into them.

References:

1. Sega GA. 1984. A review of the genetic effects of ethyl methanesulfonate. Mutat Res 134:113-42.

2. Kilbey BJ. 1975. Mutagenesis in yeast. Methods Cell Biol 12:209-31.

3. Vyas VK, Barrasa MI, Fink GR. 2015. A Candida albicans CRISPR system permits genetic engineering of essential genes and gene families. Sci Adv 1:e1500248.

4. Skrzypek MS, Binkley J, Binkley G, Miyasato SR, Simison M, Sherlock G. 2017. The Candida Genome Database (CGD): incorporation of Assembly 22, systematic identifiers and visualization of high throughput sequencing data. Nucleic Acids Research 45:D592-D596.

5. Adli M. 2018. The CRISPR tool kit for genome editing and beyond. Nat Commun 9:1911.

6. Nadakuduti SS, Enciso-Rodriguez F. 2020. Advances in Genome Editing With CRISPR Systems and Transformation Technologies for Plant DNA Manipulation. Front Plant Sci 11:637159.

7. Ledford H. 2016. The Unsung Heroes of Crispr. Nature 535:342-344.

8. Lander ES. 2016. The Heroes of CRISPR. Cell 164:18-28.

9. Adli M. 2018. The CRISPR tool kit for genome editing and beyond. Nature Communications 9.

10. Vyas VK, Bushkin GG, Bernstein DA, Getz MA, Sewastianik M, Barrasa MI, Bartel DP, Fink GR. 2018. New CRISPR Mutagenesis Strategies Reveal Variation in Repair Mechanisms among Fungi. Msphere 3.

11. Skrzypek MS, Binkley J, Sherlock G. 2016. How to Use the Candida Genome Database. Methods Mol Biol 1356:3-15.

12. Evans BA, Smith OL, Pickerill ES, York MK, Buenconsejo KJP, Chambers AE, Bernstein DA. 2018. Restriction digest screening facilitates efficient detection of site-directed mutations introduced by CRISPR in C. albicans UME6. PeerJ 6:e4920.

13. Evans BA, Pickerill ES, Vyas VK, Bernstein DA. 2018. CRISPR-mediated Genome Editing of the Human Fungal Pathogen Candida albicans. J Vis Exp doi:10.3791/58764.

**Appendix 2**


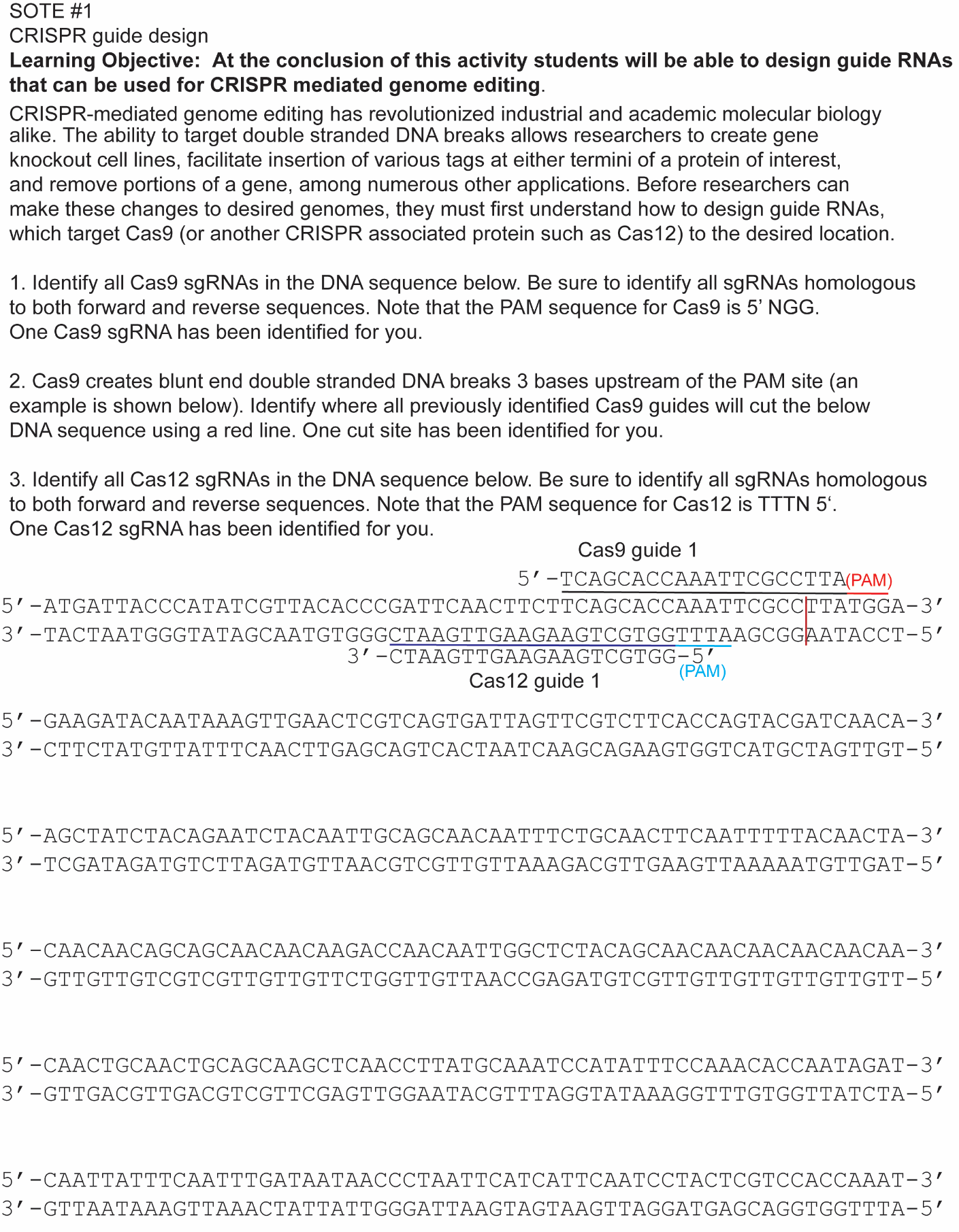


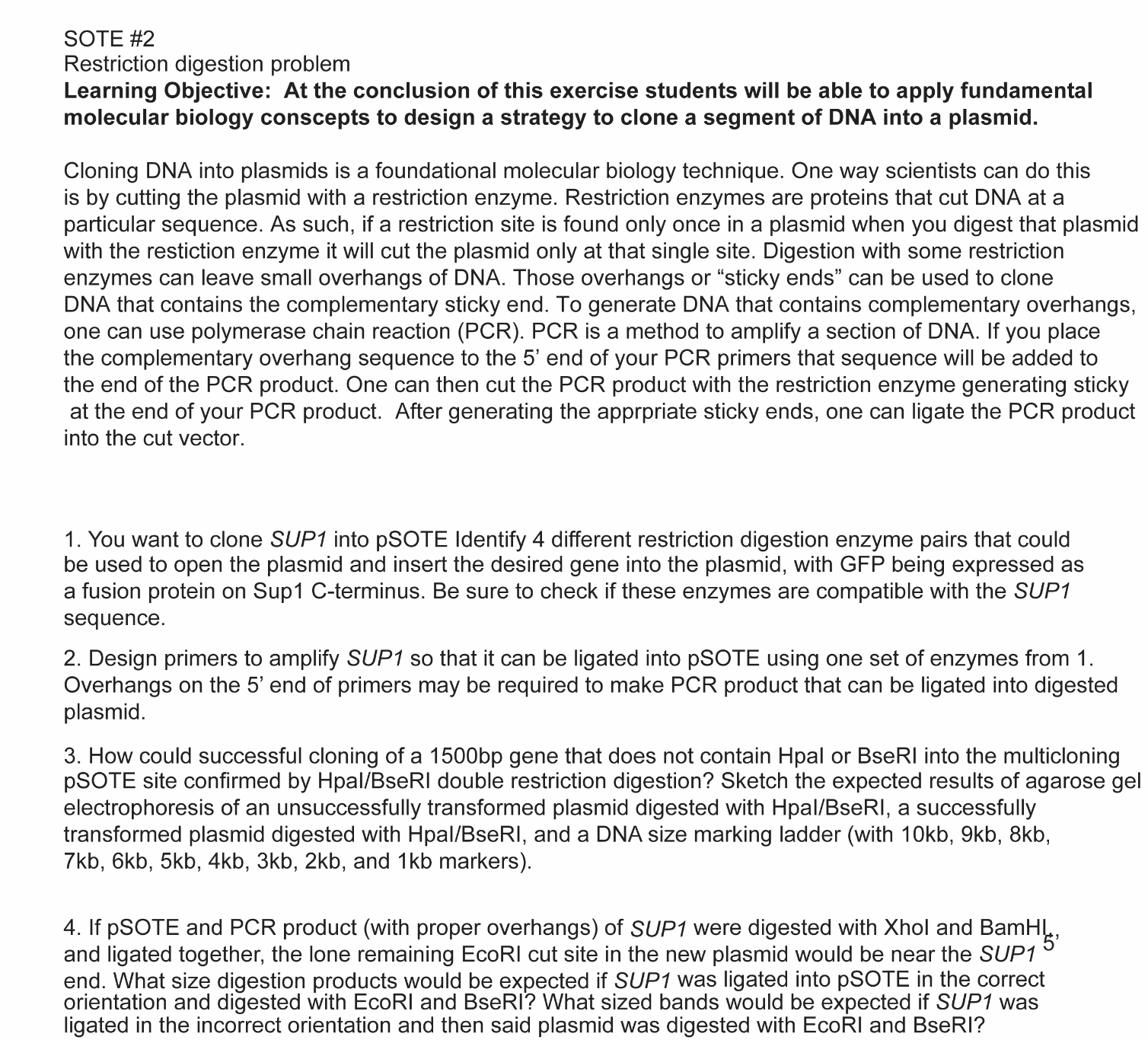


­­­­
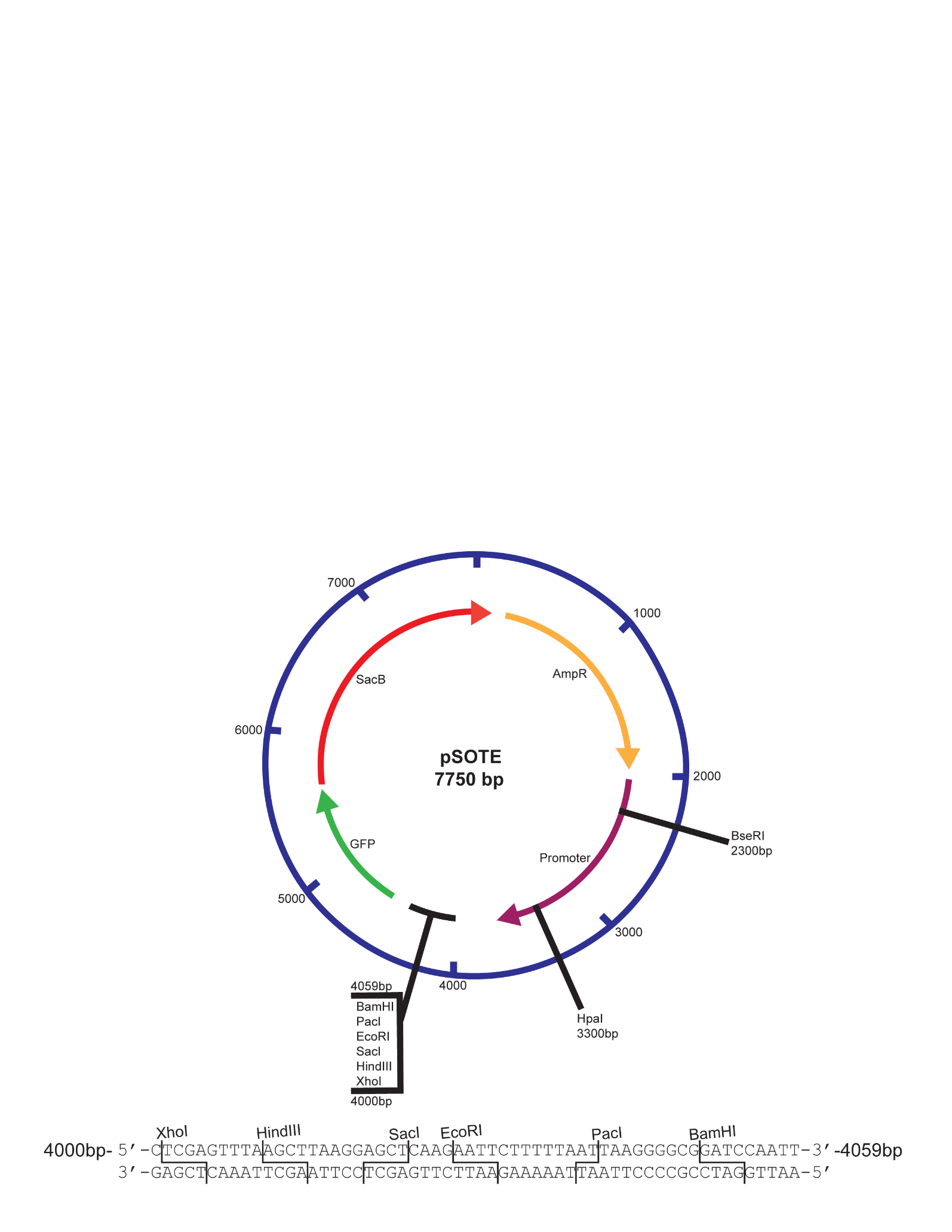


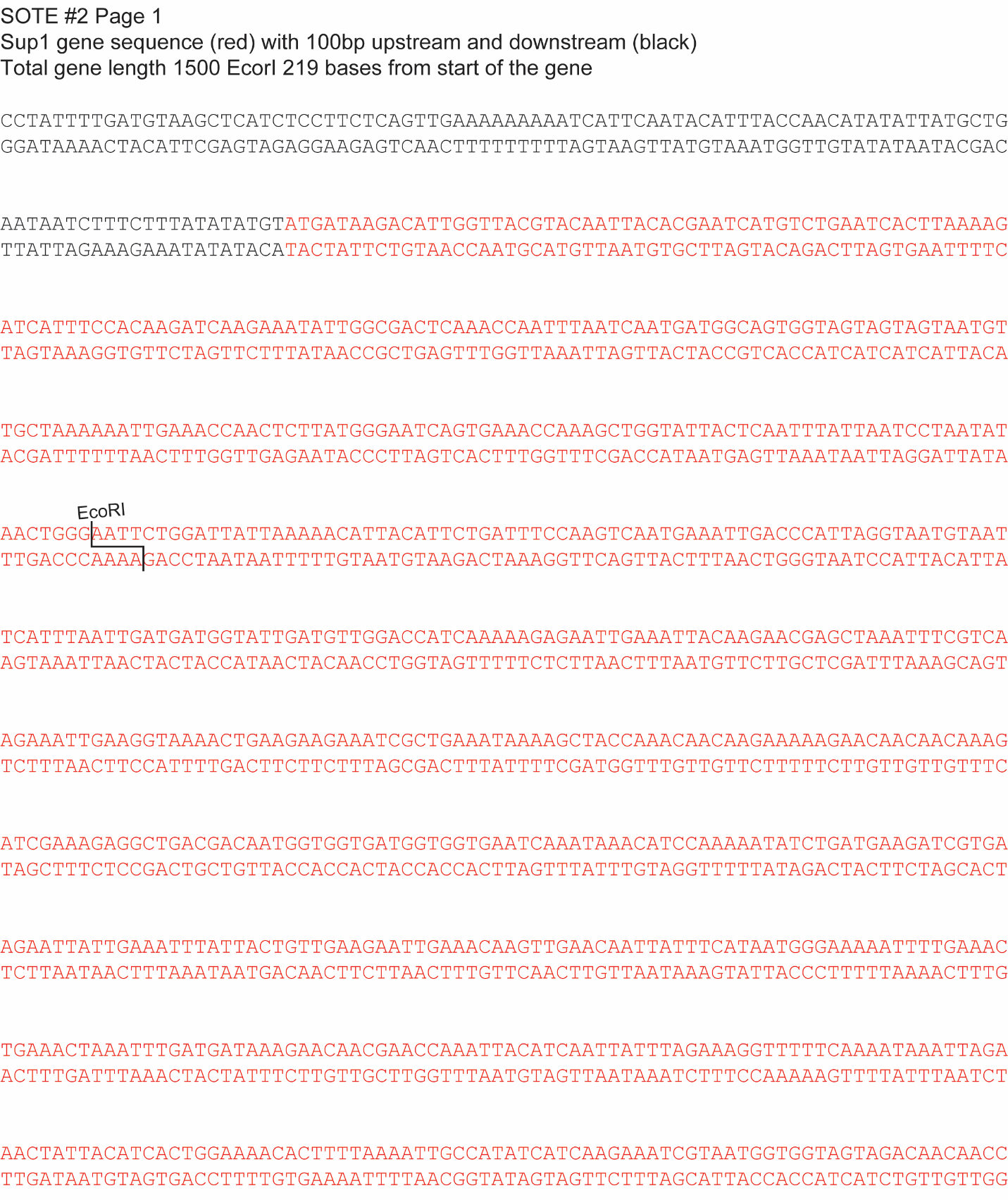


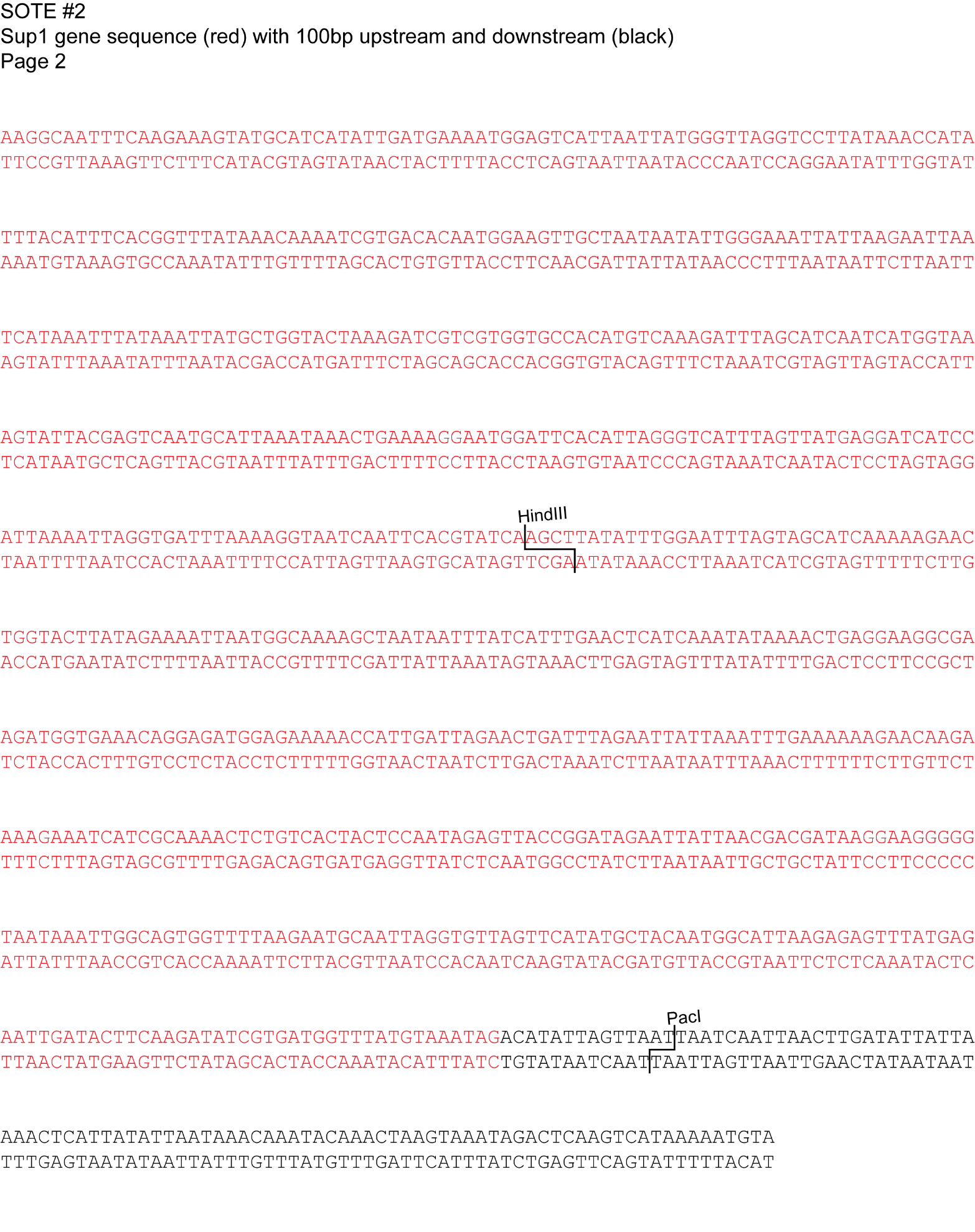


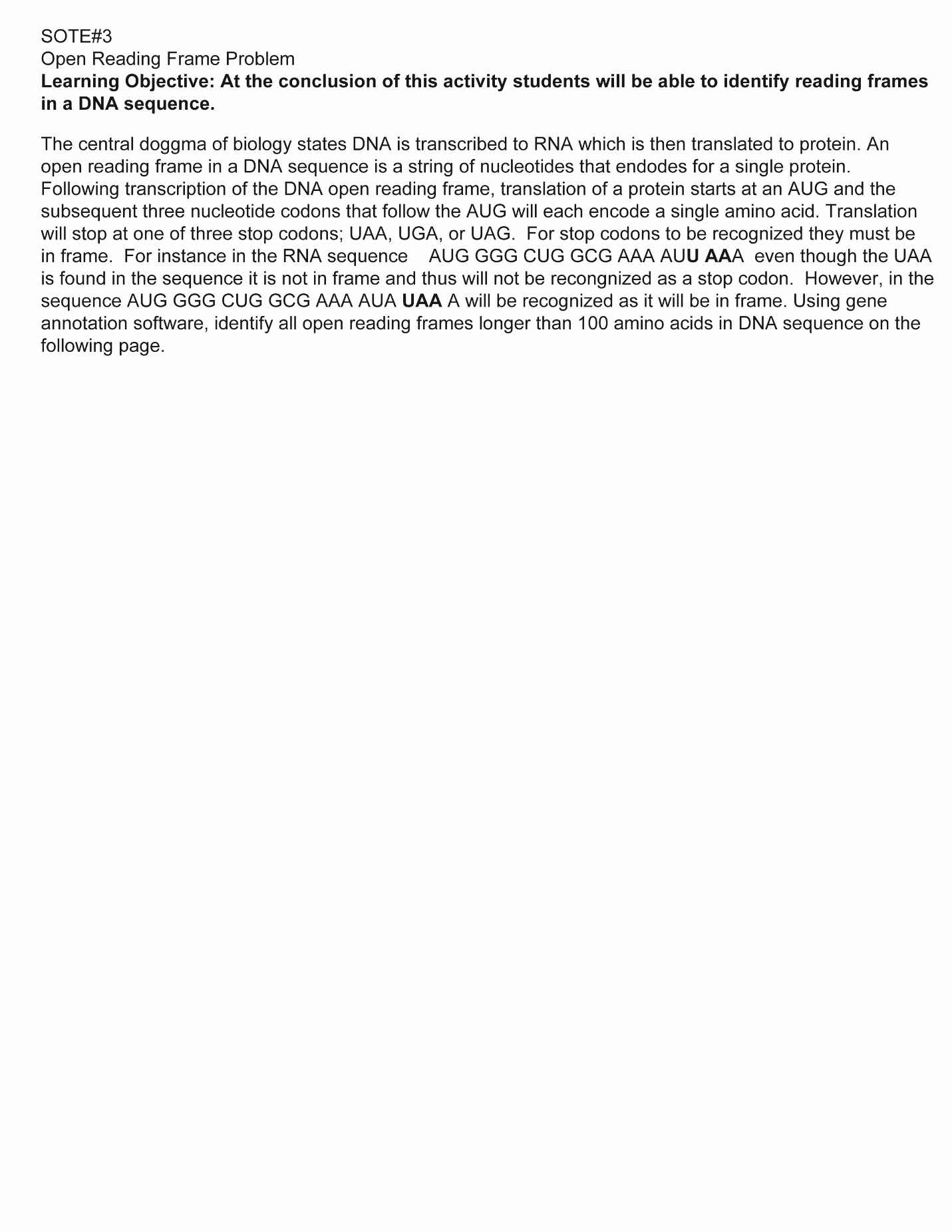


SOTE#3 DNA Sequence
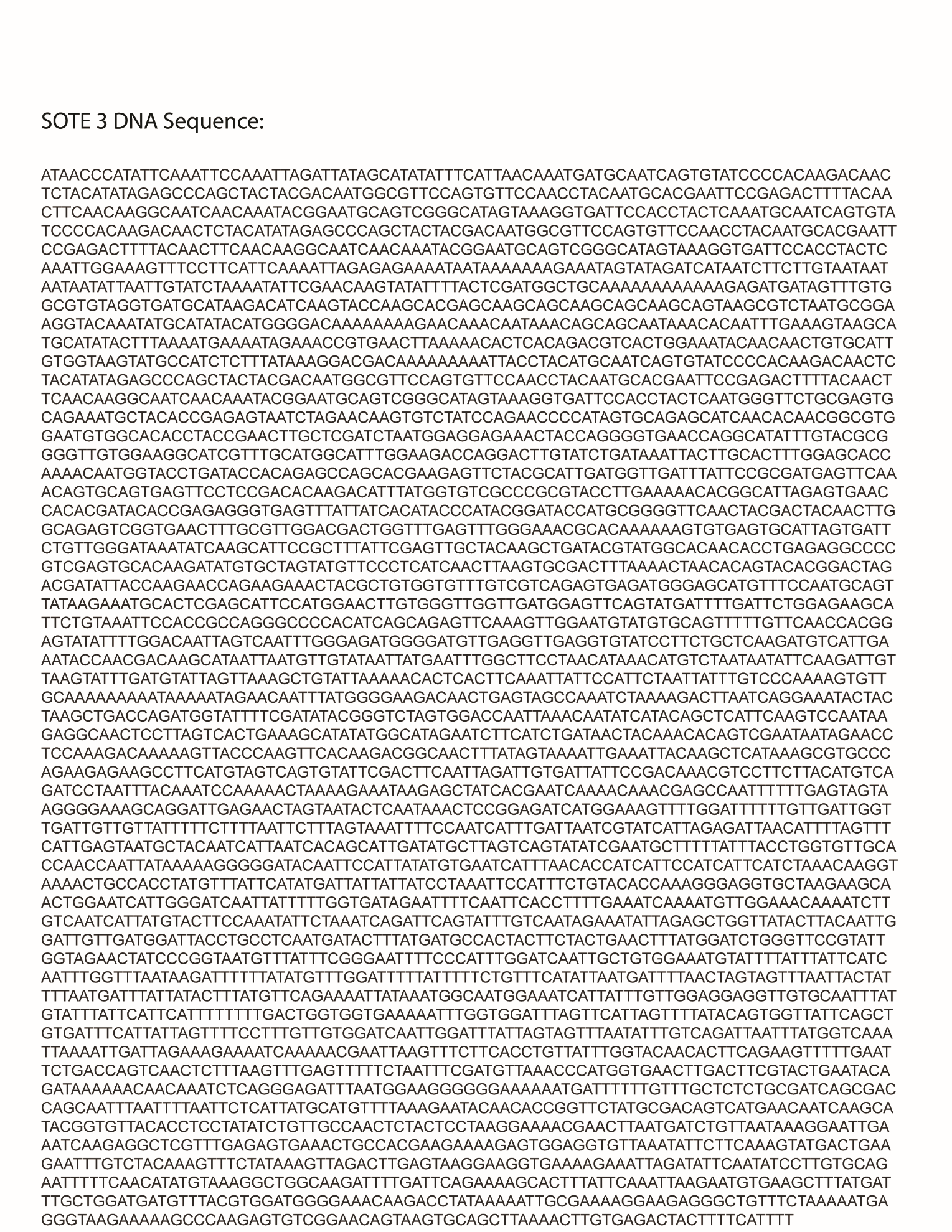


SOTE Answer Key

**
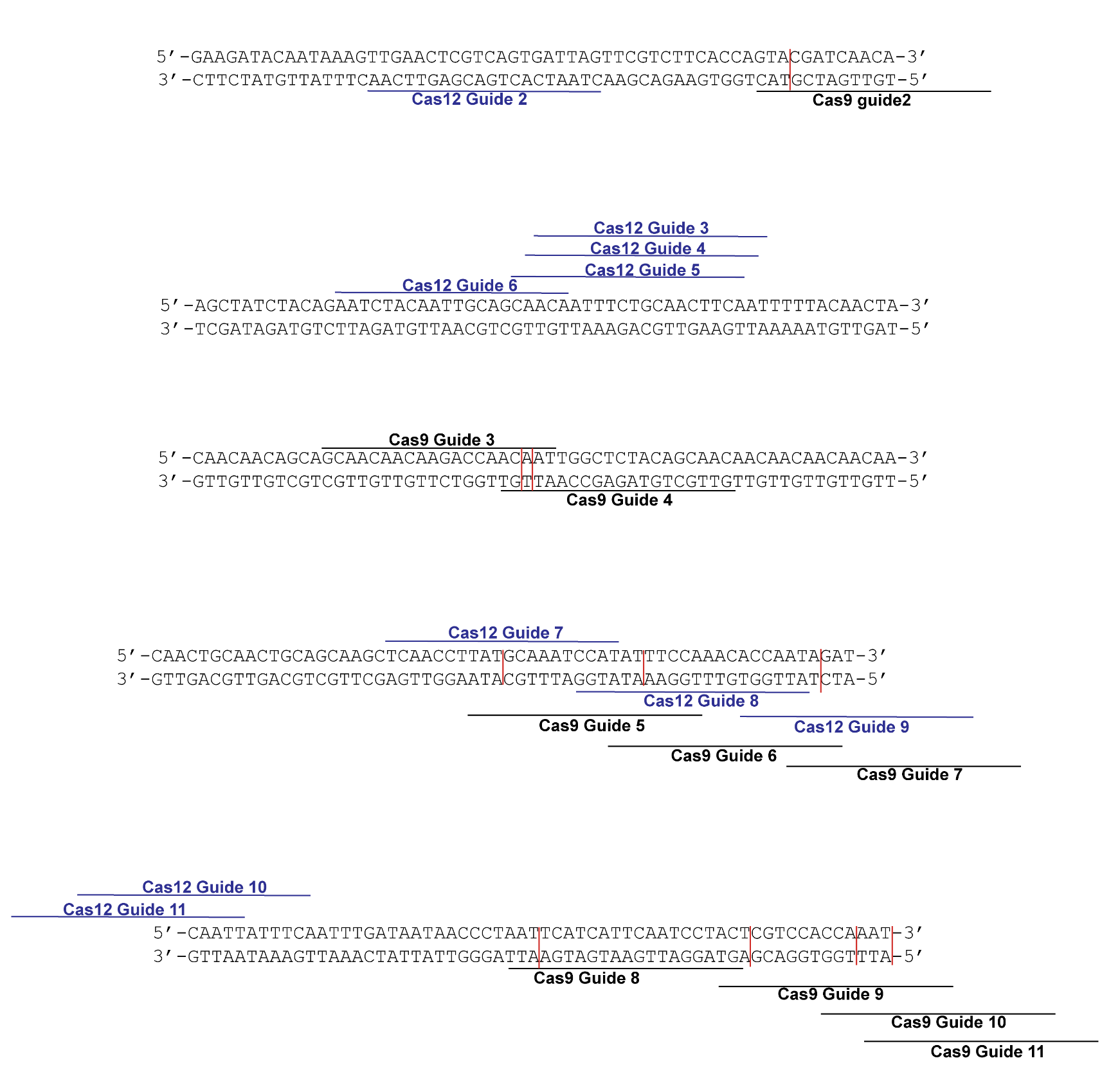
SOTE#1 Answer Key**

| Guide | Sequence 5’ -> 3’ |
| --- | --- |
| Cas9 Guide 2 forward | NNNNNNNNTGTTGATCGTAC |
| Cas9 Guide 2 reverse | GTACGATCAACANNNNNNNN |
| Cas9 Guide 3 forward | GCAACAACAAGACCAACAAT |
| Cas9 Guide 3 reverse | ATTGTTGGTCTTGTTGTTGC |
| Cas9 Guide4 forward | GTTGCTGTAGAGCCAATTGT |
| Cas9 Guide 4 reverse | ACAATTGGCTCTACAGCAAC |
| Cas9 Guide 5 forward | TGGAAATATGGATTTGCATA |
| Cas9 Guide 5 reverse | TATGCAAATCCATATTTCCA |
| Cas9 Guide 6 forward | TCTATTGGTGTTTGGAAATA |
| Cas9 Guide 6 reverse | TATTTCCAAACACCAATAGA |
| Cas9 Guide 7 forward | NNNNNNNNNNNNNNATCTAT |
| Cas9 Guide 7 reverse | ATAGATNNNNNNNNNNNNNN |
| Cas9 Guide 8 forward | GTAGGATTGAATGATGAATT |
| Cas9 Guide 8 reverse | AATTCATCATTCAATCCTAC |
| Cas9 Guide 9 forward | NNNNNATTTGGTGGACGAGT |
| Cas9 Guide 9 reverse | ACTCGTCCACCAAATNNNNN |
| Cas9 Guide 10 forward | NNNNNNNNNNNNNNATTTGG |
| Cas9 Guide 10 reverse | CCAAACNNNNNNNNNNNNNN |
| Cas9 Guide 11 forward | NNNNNNNNNNNNNNNNNATT |
| Cas9 Guide 11 reverse | AATNNNNNNNNNNNNNNNNN |
| Cas12 Guide 2 forward | CTAATCACTGACGAGTTCAA |
| Cas12 Guide 2 reverse | TTGAACTCGTCAGTGATTAG |
| Cas12 Guide 3 forward | ACAATTTCTGCAACTTCAAT |
| Cas12 Guide 3 reverse | ATTGAAGTTGCAGAAATTGT |
| Cas12 Guide4 forward | AACAATTTCTGCAACTTCAA |
| Cas12 Guide 4 reverse | TTGAAGTTGCAGAAATTGTT |
| Cas12 Guide 5 forward | CAACAATTTCTGCAACTTCA |
| Cas12 Guide 5 reverse | TGAAGTTGCAGAAATTGTTG |
| Cas12 Guide 6 forward | AATCTACAATTGCAGCAACA |
| Cas12 Guide 6 reverse | TGTTGCTGCAATTGTAGATT |
| Cas12 Guide 7 forward | TCAACCTTATGCAAATCCAT |
| Cas12 Guide 7 reverse | ATGGATTTGCATAAGGTTGA |
| Cas12 Guide 8 forward | ATTGGTGTTTGGAAATATGG |
| Cas12 Guide 8 reverse | CCATATTTCCAAACACCAAT |
| Cas12 Guide 9 forward | NNNNNNNNNNTGGTTATCTA |
| Cas12 Guide 9 reverse | TAGATAACCANNNNNNNNNN |
| Cas12 Guide 10 forward | NNNNNNNNNCAATTATTTCA |
| Cas12 Guide 10 reverse | TGAAATAATTGNNNNNNNNN |
| Cas12 Guide 11 forward | NNNNNNNNNNNNNNNCAATT |
| Cas12 Guide 11 reverse | AATTGNNNNNNNNNNNNNNN |

**SOTE#2 Answer Key**

1) Possible Answers

a. XhoI and BamHI
b. Xho1 and Sac1

c. Sac1 and BamH1

d. Sac1 and Pac1

e. Xho1 and Pac1

f. Pac1 and BamH1

2) Primers for *SUP1* amplification must contain “sticky ends” (uppercase) matching the ends left by restriction site and ~20 bp of homology to *SUP1* lowercase. Additional bases may be needed to generate the sticky ends depending upon the restriction enzyme but this is not part of the learning objective for this exercise.

a. XhoI and BamHI

Fr CTCGAGatgataagacattggttacg

Rv GGATCCctatttacataaaccatcac

b. Xho1 and Sac1

Fr CTCGAGatgataagacattggttacg

Rv GAGCTCctatttacataaaccatcac

c. Sac1 and BamH1

Fr GAGCTCatgataagacattggttacg

Rv GGATCCctatttacataaaccatcac

d. Sac1 and Pac1

Fr GAGCTCatgataagacattggttacg

Rv TTAATTAActatttacataaaccatcac

e. Xho1 and Pac1

Fr CTCGAGatgataagacattggttacg

Rv TTAATTAActatttacataaaccatcac

f. Pac1 and BamH1

Fr TTAATTAAatgataagacattggttacg

Rv GGATCCctatttacataaaccatcac

3) Adding a 1500 bp gene into the plasmid will result in a longer sequence, which will not travel as far on an agarose gel as a digested plasmid with no insert.


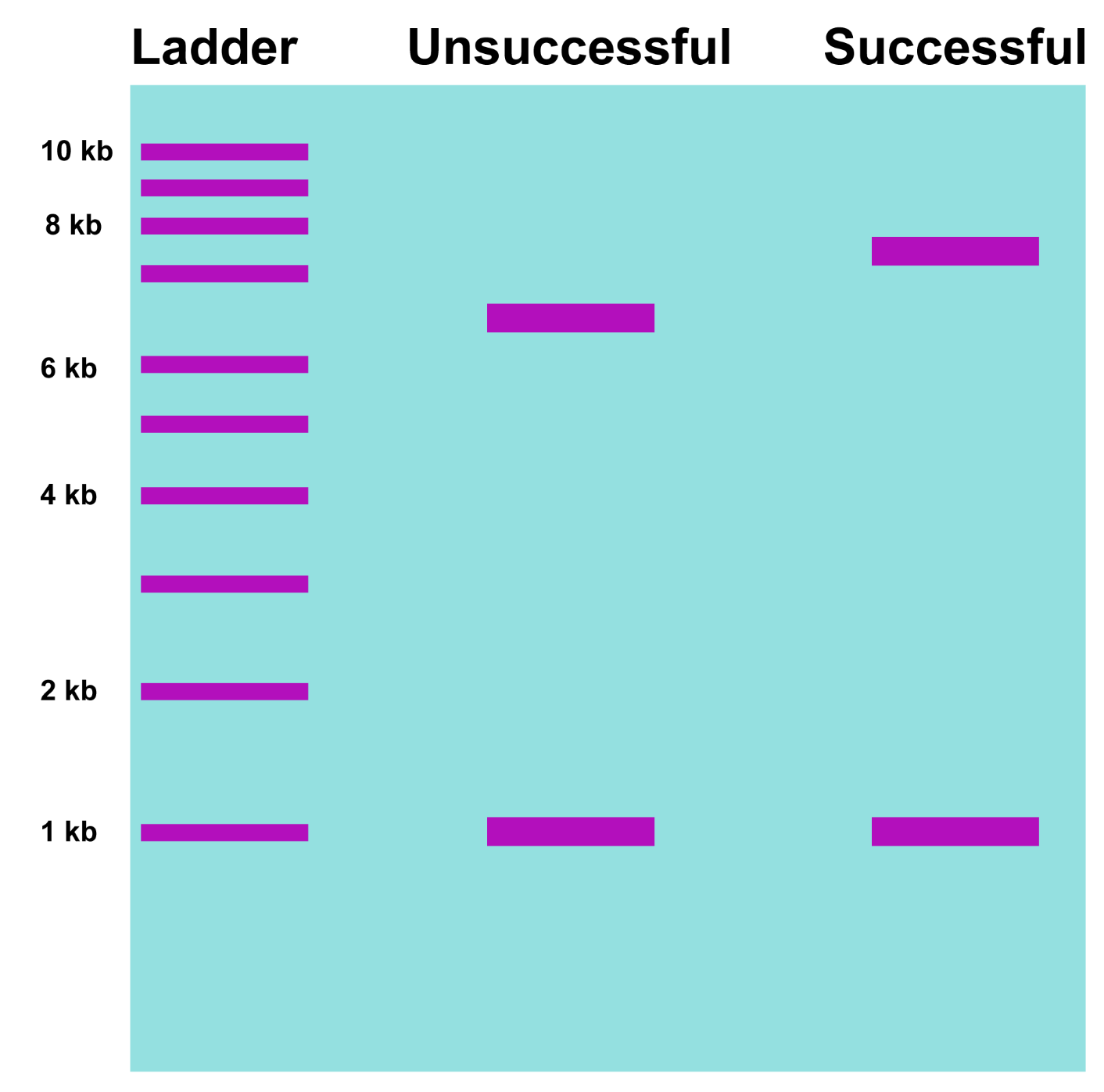


4) If *SUP1* was inserted in the correct orientation, digestion with EcoRI and BseRI will yield bands approximately 1,924 and 7,282 bp bands. If *SUP1* was inserted incorrectly, digestion will yield approximately 2,981 and 6,275 bp bands.

**SOTE#3 Answer Key**

There are 6 ORFs longer than 100 amino acids.

1. 53 – 496
2. 499 – 834
3. 828 – 2138
4. 2368 – 2802
5. 2941 – 3870
6. 4171 – 4638

Red indicates ORFs in the 5’ 🡪 3’ direction

Green indicates ORFs in the 3’ 🡪 5’ direction

Purple indicates where two ORFs overlap

ataacccatattcaaattccaaattagattatagcatatatttcattaacaaatgatgcaatcagtgtatccccacaagacaactctacatatagagcccagctactacgacaatggcgttccagtgttccaacctacaatgcacgaattccgagacttttacaacttcaacaaggcaatcaacaaatacggaatgcagtcgggcatagtaaaggtgattccacctactcaaatgcaatcagtgtatccccacaagacaactctacatatagagcccagctactacgacaatggcgttccagtgttccaacctacaatgcacgaattccgagacttttacaacttcaacaaggcaatcaacaaatacggaatgcagtcgggcatagtaaaggtgattccacctactcaaattggaaagtttccttcattcaaaattagagagaaaataataaaaaaagaaatagtatagatcataatcttcttgtaataataataatattaattgtatctaaaatattcgaacaagtatattttactcgatggctgcaaaaaaaaaaaagagatgatagtttgtggcgtgtaggtgatgcataagacatcaagtaccaagcacgagcaagcagcaagcagcaagcagtaagcgtctaatgcggaaggtacaaatatgcatatacatggggacaaaaaaaagaacaaacaataaacagcagcaataaacacaatttgaaagtaagcatgcatatactttaaaatgaaaatagaaaccgtgaacttaaaaacactcacagacgtcactggaaatacaacaactgtgcattgtggtaagtatgccatctctttataaaggacgacaaaaaaaaattacctacatgcaatcagtgtatccccacaagacaactctacatatagagcccagctactacgacaatggcgttccagtgttccaacctacaatgcacgaattccgagacttttacaacttcaacaaggcaatcaacaaatacggaatgcagtcgggcatagtaaaggtgattccacctactcaatgggttctgcgagtgcagaaatgctacaccgagagtaatctagaacaagtgtctatccagaaccccatagtgcagagcatcaacacaacggcgtggaatgtggcacacctaccgaacttgctcgatctaatggaggagaaactaccaggggtgaaccaggcatatttgtacgcggggttgtggaaggcatcgtttgcatggcatttggaagaccaggacttgtatctgataaattacttgcactttggagcaccaaaacaatggtacctgataccacagagccagcacgaagagttctacgcattgatggttgatttattccgcgatgagttcaaacagtgcagtgagttcctccgacacaagacatttatggtgtcgcccgcgtaccttgaaaaacacggcattagagtgaaccacacgatacaccgagagggtgagtttattatcacatacccatacggataccatgcggggttcaactacgactacaacttggcagagtcggtgaactttgcgttggacgactggtttgagtttgggaaacgcacaaaaaagtgtgagtgcattagtgattctgttgggataaatatcaagcattccgctttattcgagttgctacaagctgatacgtatggcacaacacctgagaggccccgtcgagtgcacaagatatgtgctagtatgttccctcatcaacttaagtgcgactttaaaactaacacagtacacggactagacgatattaccaagaaccagaagaaactacgctgtggtgtttgtcgtcagagtgagatgggagcatgtttccaatgcagttataagaaatgcactcgagcattccatggaacttgtgggttggttgatggagttcagtatgattttgattctggagaagcattctgtaaattccaccgccagggccccacatcagcagagttcaaagttggaatgtatgtgcagtttttgttcaaccacggagtatattttggacaattagtcaatttgggagatggggatgttgaggttgaggtgtatccttctgctcaagatgtcattgaaataccaacgacaagcataattaatgttgtataattatgaatttggcttcctaacataaacatgtctaataatattcaagattgttaagtatttgatgtattagttaaagctgtattaaaaacactcacttcaaattattccattctaattatttgtcccaaaagtgttgcaaaaaaaaataaaaatagaacaatttatggggaagacaactgagtagccaaatctaaaagacttaatcaggaaatactactaagctgaccagatggtattttcgatatacgggtctagtggaccaattaaacaatatcatacagctcattcaagtccaataagaggcaactccttagtcactgaaagcatatatggcatagaatcttcatctgataactacaaacacagtcgaataatagaacctccaaagacaaaaagttacccaagttcacaagacggcaactttatagtaaaattgaaattacaagctcataaagcgtgcccagaagagaagccttcatgtagtcagtgtattcgacttcaattagattgtgattattccgacaaacgtccttcttacatgtcagatcctaatttacaaatccaaaaactaaaagaaataagagctatcacgaatcaaaacaaacgagccaattttttgagtagtaaggggaaagcaggattgagaactagtaatactcaataaactccggagatcatggaaagttttggattttttgttgattggttgattgttgttatttttcttttaattctttagtaaattttccaatcatttgattaatcgtatcattagagattaacattttagtttcattgagtaatgctacaatcattaatcacagcattgatatgcttagtcagtatatcgaatgctttttatttacctggtgttgcaccaaccaattataaaaagggggatacaattccattatatgtgaatcatttaacaccatcattccatcattcatctaaacaaggtaaaactgccacctatgtttattcatatgattattattatcctaaattccatttctgtacaccaaagggaggtgctaagaagcaactggaatcattgggatcaattatttttggtgatagaattttcaattcaccttttgaaatcaaaatgttggaaacaaaatcttgtcaatcattatgtacttccaaatattctaaatcagattcagtatttgtcaatagaaatattagagctggttatacttacaattggattgttgatggattacctgcctcaatgatactttatgatgccactacttctactgaactttatggatctgggttccgtattggtagaactatcccggtaatgtttatttcgggaattttcccatttggatcaattgctgtggaaatgtattttatttattcatcaatttggtttaataagattttttatatgtttggatttttatttttctgtttcatattaatgattttaactagtagtttaattactattttaatgatttattatactttatgttcagaaaattataaatggcaatggaaatcattatttgttggaggaggttgtgcaatttatgtatttattcattcattttttttgactggtggtgaaaaatttggtggatttagttcattagttttatacagtggttattcagctgtgatttcattattagttttcctttgttgtggatcaattggatttattagtagtttaatatttgtcagattaatttatggtcaaattaaaattgattagaaagaaaatcaaaaacgaattaagtttcttcacctgttatttggtacaacacttcagaagtttttgaattctgaccagtcaactctttaagtttgagtttttctaatttcgatgttaaacccatggtgaacttgacttcgtactgaatacagataaaaaacaacaaatctcagggagatttaatggaaggggggaaaaaatgattttttgtttgctctctgcgatcagcgaccagcaatttaattttaattctcattatgcatgttttaaagaatacaacaccggttctatgcgacagtcatgaacaatcaagcatacggtgttacacctcctatatctgttgccaactctactcctaaggaaaacgaacttaatgatctgttaataaaggaattgaaatcaagaggctcgtttgagagtgaaactgccacgaagaaaagagtggaggtgttaaatattcttcaaagtatgactgaagaatttgtctacaaagtttctataaagttagacttgagtaaggaaggtgaaaagaaattagatattcaatatccttgtgcagaatttttcaacatatgtaaaggctggcaagattttgattcagaaaagcactttattcaaattaagaatgtgaagctttatgatttgctggatgatgtttacgtggatggggaaacaagacctataaaaattgcgaaaaggaagagggctgtttctaaaaatgagggtaagaaaaagcccaagagtgtcggaacagtaagtgcagcttaaaacttgtgagactacttttcatttt

Common student challenges when performing these SOTES

SOTE#1: Students must remember that the 5’-NGG sites can be found on both strands but they are always 5’-NGG-3’.

SOTE#2: Students must take notice of which restrictions sites are found within the gene they are cloning into the vector. Using those sites as your cloning sites will lead to cleavage of the gene and cloning of a gene fragment as opposed to the whole gene.

SUP1 Gene sequence

ATGATAAGACATTGGTTACGTACAATTACACGAATCATGTCTGAATCACTTAAAAGATCATTTCCACAAGATCAAGAAATATTGGCGACTCAAACCAATTTAATCAATGATGGCAGTGGTAGTAGTAGTAATGTTGCTAAAAAATTGAAACCAACTCTTATGGGAATCAGTGAAACCAAAGCTGGTATTACTCAATTTATTAATCCTAATATAACTGGGAATTCTGGATTATTAAAAACATTACATTCTGATTTCCAAGTCAATGAAATTGACCCATTAGGTAATGTAATTCATTTAATTGATGATGGTATTGATGTTGGACCATCAAAAAGAGAATTGAAATTACAAGAACGAGCTAAATTTCGTCAAGAAATTGAAGGTAAAACTGAAGAAGAAATCGCTGAAATAAAAGCTACCAAACAACAAGAAAAAGAACAACAACAAAGATCGAAAGAGGCTGACGACAATGGTGGTGATGGTGGTGAATCAAATAAACATCCAAAAATATCTGATGAAGATCGTGAAGAATTATTGAAATTTATTACTGTTGAAGAATTGAAACAAGTTGAACAATTATTTCATAATGGGAAAAATTTTGAAACTGAAACTAAATTTGATGATAAAGAACAACGAACCAAATTACATCAATTATTTAGAAAGGTTTTTCAAAATAAATTAGAAACTATTACATCACTGGAAAACACTTTTAAAATTGCCATATCATCAAGAAATCGTAATGGTGGTAGTAGACAACAACCAAGGCAATTTCAAGAAAGTATGCATCATATTGATGAAAATGGAGTCATTAATTATGGGTTAGGTCCTTATAAACCATATTTACATTTCACGGTTTATAAACAAAATCGTGACACAATGGAAGTTGCTAATAATATTGGGAAATTATTAAGAATTAATCATAAATTTATAAATTATGCTGGTACTAAAGATCGTCGTGGTGCCACATGTCAAAGATTTAGCATCAATCATGGTAAAGTATTACGAGTCAATGCATTAAATAAACTGAAAAGGAATGGATTCACATTAGGGTCATTTAGTTATGAGGATCATCCATTAAAATTAGGTGATTTAAAAGGTAATCAATTCACGTATCAAGCTTATATTTGGAATTTAGTAGCATCAAAAAGAACTGGTACTTATAGAAAATTAATGGCAAAAGCTAATAATTTATCATTTGAACTCATCAAATATAAAACTGAGGAAGGCGAAGATGGTGAAACAGGAGATGGAGAAAAACCATTGATTAGAACTGATTTAGAATTATTAAATTTGAAAAAAGAACAAGAAAAGAAATCATCGCAAAACTCTGTCACTACTCCAATAGAGTTACCGGATAGAATTATTAACGACGATAAGGAAGGGGGTAATAAATTGGCAGTGGTTTTAAGAATGCAATTAGGTGTTAGTTCATATGCTACAATGGCATTAAGAGAGTTTATGAGAATTGATACTTCAAGATATCGTGATGGTTTATGTAAATAG

SOTE#3: Students must remember that open reading frames can go in both directions and they can overlap. Furthermore, open reading frames do not need to be in frame with one another.
